# Genome-wide identification and expression profiling of basic leucine zipper transcription factors following abiotic stresses in potato (*Solanum tuberosum* L.)

**DOI:** 10.1371/journal.pone.0247864

**Published:** 2021-03-12

**Authors:** Pankaj Kumar, Pankaj Kumar, Dixit Sharma, Shailender Kumar Verma, Dennis Halterman, Arun Kumar

**Affiliations:** 1 School of Agricultural Biotechnology, Punjab Agricultural University, Ludhiana, India; 2 Biotechnology Division, CSIR-Institute of Himalayan Bioresource Technology, Palampur, Himachal Pradesh, India; 3 Centre for Computational Biology and Bioinformatics, School of Life Sciences, Central University of Himachal Pradesh, Kangra, Himachal Pradesh, India; 4 U.S. Department of Agriculture-Agricultural Research Service, Madison, Wisconsin, United States of America; 5 Academy of Scientific and Innovative Research, Ghaziabad, Uttar Pradesh, India; Government College University Faisalabad, PAKISTAN

## Abstract

Potato (*Solanum tuberosum* L.) is an important food crop that is grown and consumed worldwide. The growth and productivity of this crop are severely affected by various abiotic stresses. Basic leucine zipper (bZIP) transcription factors (TFs) in plants are well known for their function during growth and development. However, systematic and in-depth identification and functional characterization of the bZIP gene family of potato is lacking. In the current study, we identified a total of 90 bZIPs (*StbZIP*) distributed on 12 linkage groups of potato. Based on the previous functional annotation and classification of bZIPs in *Arabidopsis*, wheat, and rice, a phylogenetic tree of potato *bZIPs* was constructed and genes were categorized into various functional groups (A to I, S, and U) as previously annotated in *Arabidopsis thaliana*. Analyses of the transcript sequence (RNA-seq) data led to identifying a total of 18 candidate *StbZIPs* [four in roots, eight in the tuber, six in mesocarp and endocarp] that were expressed in a tissue-specific manner. Differential expression analysis under the various abiotic conditions (salt, mannitol, water, and heat stress) and treatment with phytohormones (ABA, GA, IAA, and BAP) led to the identification of forty-two [thirteen under salt stress, two under mannitol stress, ten under water stress, and eighteen under heat stress], and eleven [eight and three *StbZIPs* upon treatment with ABA, and IAA, respectively] candidate *StbZIPs*, respectively. Using sequence information of candidate *StbZIPs*, a total of 22 SSR markers were also identified in this study. In conclusion, the genome-wide identification analysis coupled with RNA-Seq expression data led to identifying candidate *StbZIPs*, which are dysregulated, and may play a pivotal role under various abiotic stress conditions. This study will pave the way for future functional studies using forward and reverse genetics to improve abiotic stress tolerance in potato.

## Introduction

One of the biggest challenges of current times is to feed the ever-growing population amidst a changing climate with limited available resources. Abiotic stresses such as salinity, water (drought and flooding), and heat are a few of the many factors that directly impact the agronomical traits and yield of crops [1]. Potato (*Solanum tuberosum* L.) is one of the most important food crops, third-largest after rice and wheat in terms of human consumption [2]. This crop can also be used commercially as a health food because it is rich in antioxidants, minerals, and dietary fibers [3]. However, as a consequence of global warming, a continuous increase in temperature leads to multiple stresses, including heat and salinity. Consequently, excess reactive oxygen species (ROS) are generated inside the cells, which, if not scavenged, can lead to severe oxidative stress to plants [3]. ROS can be scavenged using an array of enzymatic [such as superoxide dismutase (SOD), ascorbate peroxidase (APX), and catalase] [3] and non-enzymatic mechanisms (glutathione, α-tocopherol, carotenoids, flavonoids) [3–5]. Impaired function or decreased activity of any of these ROS scavenging activities can also have a devastating impact on the plant’s growth and yield [6]. Stress also affects the quality of potato tubers by altering sugar and solid content [7]. A limited understanding of heat and salt tolerance regulation mechanisms through TFs (transcription factors) hinders elite cultivars’ development with higher stress tolerance [7, 8]. The complete genome sequence data of potato (haploid genome content of 840 Mb) offers an excellent opportunity to explore gene families, their downstream pathways, and regulation mechanisms under stress conditions.

In the current study, we mined and functionally annotated the basic leucine zipper family (bZIP) of TFs in potato using its genome information and previous annotation [9–11]. Generally, the bZIP proteins contain an essential region that consists of a DNA-binding part and a leucine zipper dimerization motif [9–12]. Structurally, the bZIP family is highly conserved among the species with signature sequences of the domains (60–80 amino acids long). These bZIPs can be subdivided further into two essential parts depending on their structure: the basic region and the leucine zipper region. The basic region has an invariant motif N-X7-R / K-X9, which links nuclear localization to the target DNA [10, 12]. The leucine zipper region consists of Leu heptad repeats, and often with Ile, Val, Phe, or Met [9–12]. Recent findings of bZIP TFs in specific plant families have shown that they are involved in diverse biological roles in plant physiology [13–16]. The functions of bZIP TFs are well characterized in various plants during stress conditions, such as salt, heat, and hormone signaling [16–20]. To date, genome-wide analysis has led to the identification of the *bZIP* gene family in several plants, including 75 in *Arabidopsis thaliana (AtbZIPs)*, 89 in *Oryza sativa* (*OsbZIPs*), 160 in *Glycine max* (*GmbZIPs*), 166 in *Sorghum bicolor* (*SbbZIPs*), 125 in *Zea mays* (*ZmbZIPs*), 136 in *Brassica napus* (*BrbZIPs*), and 370 in *Triticum aestivum* (*TabZIPs*), and 41 in *Ipomoea trifida* [10, 11, 21–23, 25, 26]. Recently, 65 *bZIPs* were identified in the potato genome using a single database source viz. Spud DB [9]. In our current study, we used the previous genome sequence databases available in SpudDB [27] as well as the latest genome assembly in the Ensembl plants database (http://plants.ensembl.org/Solanum_tuberosum) of potato for a comprehensive investigation of the *StbZIP* family that includes transcripts and genes. The present work led to the identification of 25 more *bZIPs* at the genome-wide scale.

A previous study has shown the role of *bZIP* StABF1 in inducing tolerance to salt, drought, and abscisic acid (ABA) stress in potatoes [28]. However, a detailed identification and characterization of the *StbZIP* gene family in response to abiotic and hormonal stress in potato is lacking. A thorough study of the gene expression pattern of *StbZIPs* under various stress conditions in the potato will help us to understand stress regulatory mechanisms. Here, we identified members of the *StbZIP* gene family using computational approaches, and the subfamily classification was performed based on prior annotations in *Arabidopsis* [10]. The distribution and conserved domain analysis of the *StbZIP* family were studied and validated using proteome sequence data and next-generation sequencing (NGS) data available in the public domain. Based on this information, a few candidate *StbZIPs* that are expressed under various stress conditions were identified. This research will be valuable in further functional validation of the *StbZIPs* and developing stress-tolerant elite varieties either using trans/cis-genic route or via molecular breeding programs.

## Material and methods

### Identification of bZIP transcription factors in the potato genome

A two-step identification process was performed to categorize bZIPs of potato. In the first step, the curated Hidden Markov Model profiles of the bZIP domain family, *viz*. PF00170 downloaded from Pfam [29], was used as a query to search the bZIP proteins in the potato proteome Ensembl database (http://plants.ensembl.org/index.html) using HMMER3.0 [30]. In the second step, a local BLASTp search was performed to identify the predicted potato bZIPs by HMMER3.0 from already known bZIPs in *Arabidopsis* [10], maize [24], rice [21], and barley [31] with the E-value of 0.00001. Such potential potato bZIPs were further explored with NCBI-CDD and InterproScan [32] for the existence and integrity of the bZIP domain. CDD is the conserved domain database at NCBI for the annotation of protein sequences along with the location of conserved domain footprints within the sequence. InterproScan enables extensive scanning of protein sequences against the Interpro database by predicting domains and important sites and classify them into families [32].

### Sequence alignment and phylogenetic analysis

The bZIP protein sequences from potato, rice, maize, barley, and *Arabidopsis* were aligned with gap opening and gap extension penalties of 10 and 0.1, respectively, using Clustal W. A Neighbor-Joining (NJ) method was used to develop a cladogram of all bZIP protein sequences. The associated taxa clustered together in the bootstrap test of 1000 replicas. The phylogenetic tree was constructed using MEGA 7 software, version 7 [33], and visualized through EvolView v2 [34].

### Protein properties and sequence analysis of potato bZIPs for detection of conserved motifs

The standalone Multiple Em (Expectation Maximization) for the Motif Elicitation (MEME version 4.11.2) [35] was used for the prediction of conserved motifs in StbZIPs. The limits specified for minimum and maximum width, and the maximum numbers of motifs were 1, 7, and 9, respectively, and motifs were numbered according to their order displayed by MEME [35].

### Chromosomal distribution and gene duplication study

**Fig 1:**
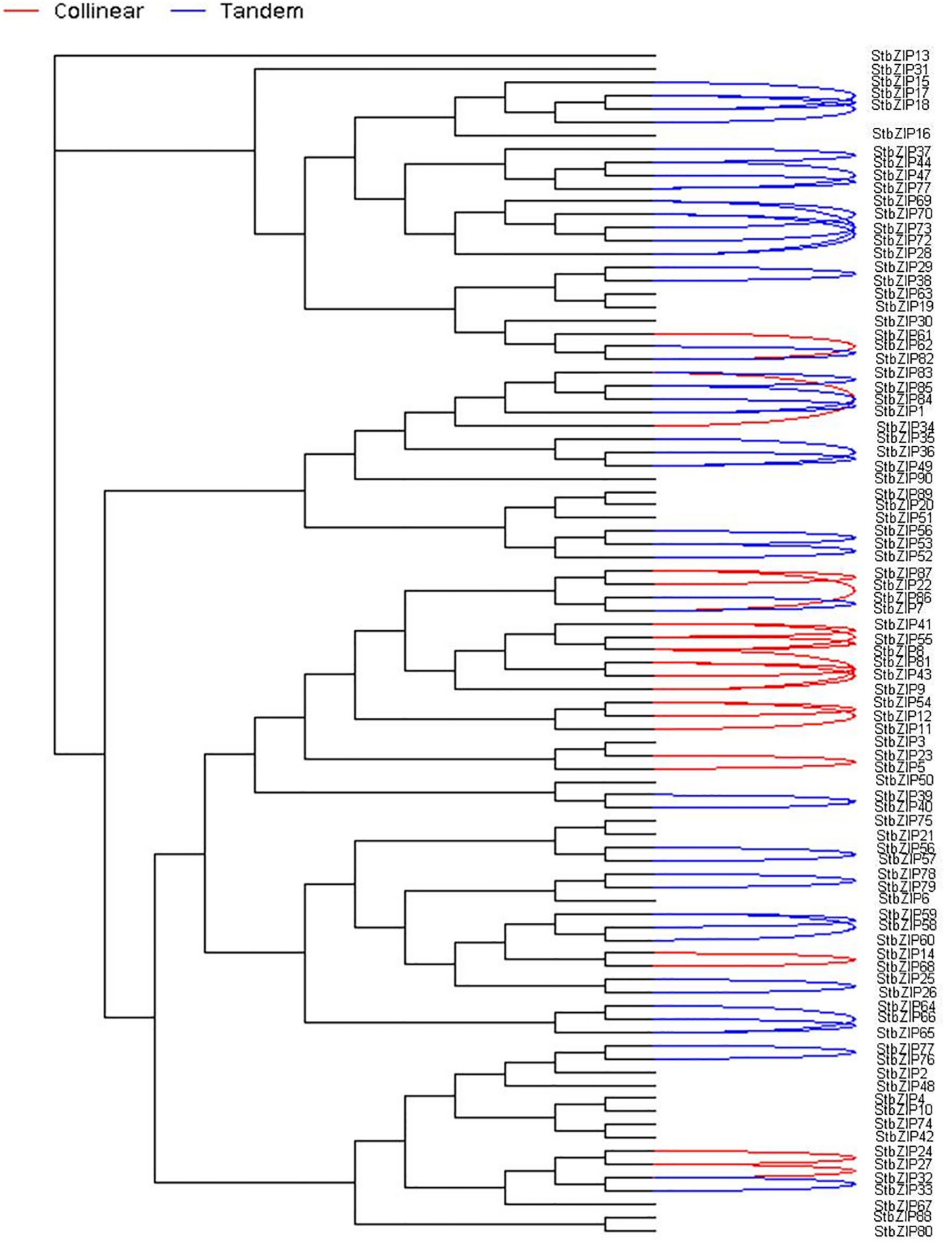
Chromosomal distribution and *StbZIP* gene duplication events in potato. The chromosomal position of each StbZIP was mapped according to the genome annotation of potato. The segmentally duplicated genes are represented as red lines, and the tandemly duplicated genes by blue lines.

The chromosomal distribution of StbZIPs was identified using the MCScanX (http:/chibba.pgml.uga.edu/mcscan2/) program to map the positions of the *StbZIP* genes within the *Solanum tuberosum* genome (plant ensemble and Spud DB (http:/solanaceae.plantbiology.msu.edu/pgsc%20download.shtml). Based on the condition of BLAST aligned query sequence (> 80 percent) of the StbZIPs, tandem and collinear gene duplications were recognized (Fig 1).

### RNA-Seq gene expression analysis

The reference genome sequence information of *Solanum tuberosum* Group Phureja DM1-3 and the FPKM values of all the representative transcripts were retrieved from Spud DB. The FPKM values were calculated by Cufflinks (v1.3.0) using v3.4 in the Spud DB database representative models for complete annotation (http://solanaceae.plantbiology.msu.edu/data/Potato_dm_v404). The relative intensity levels (heat map) and linkage hierarchical cluster analysis were performed using multiple experiments viewer (MeV 4.9.0) with differential gene expression of *StbZIPs* under various stress conditions [36–38]. Expression levels are shown as FPKM’s log 2 ratios between control and treatment samples using Pearson uncentered distance as a statistical method. The color represents the logarithmic intensity of the expressed *StbZIPs*, in color with high and low expression.

### Mining of simple sequence repeats (SSRs) from the candidate *StbZIPs*

The coding sequences of candidate *StbZIPs* were retrieved from the Ensembl plant reference database of potato, and the SSRs were predicted using MISA (http://pgrc.ipk103%20gatersleben.de/misa/) with criteria of a minimum length of six for dinucleotide repeats and five for trinucleotide repeats, tetranucleotide repeats, pentanucleotide repeats, and hexanucleotide repeats. Mix type SSRs were considered disrupted by a non-repetitive repeats length of 100 base pair sequence.

## Results and discussion

### Identification of bZIP transcription factor family in potato

**Fig 2:**
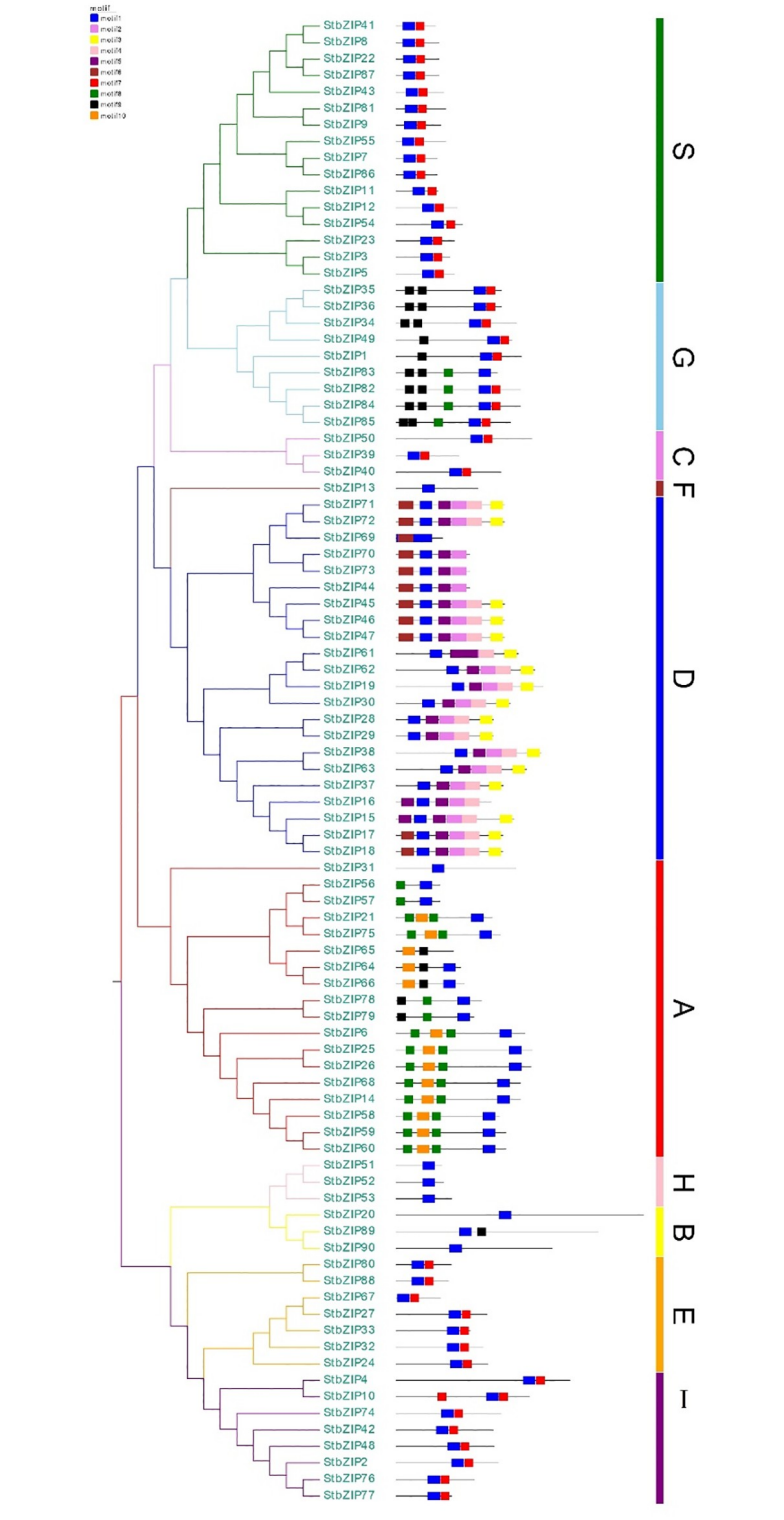
Motif and cluster analysis of StbZIPs. The conserved motifs of StbZIPs were identified using the MEME database. Based on the clustering relationship, the StbZIPs were classified into ten groups shown as A to I, and S.

The genome-wide analysis resulted in identifying a total of 90 potato StbZIPs using a combination of Hidden Markov Model (HMM) profile PF00170, a search against the whole potato proteome using Ensembl database by HMMER3.0, and BLASTp search using *Arabidopsis*, maize, rice, and barley bZIP sequences. Each potato bZIP protein was assigned a unique identifier from StbZIP1 to StbZIP90, and the information on StbZIPs is depicted in Table 1 and Fig 2. Since the cultivated potato (*Solanum tuberosum* L.) is highly heterozygous and presents challenges in genome analyses and breeding, the information derived from diploid potatoes genomic resources will aid in the identification of gene variants such as copy number variation, single nucleotide polymorphism, and genomic-enabled development of inbred diploid potatoes and improvement of cultivated tetraploid potatoes.

**Table 1:** List of ninety StbZIPs with sequence IDs, gene name, protein length, group, and domain position.

bZIP_ID	Sequence ID	Gene name	Protein Length (aa)	Group	Position *of b*ZI*P d*omain (Start-End)
StbZIP1	PGSC0003DMT400000266	PGSC0003DMG400000088	417	G	274–336
StbZIP2	PGSC0003DMT400002100	PGSC0003DMG400000799	340	I	184–242
StbZIP3	PGSC0003DMT400003516	PGSC0003DMG400001387	180	S	81–135
StbZIP4	PGSC0003DMT400004178	PGSC0003DMG400001659	577	I	421–479
StbZIP5	PGSC0003DMT400004821	PGSC0003DMG400001916	194	S	86–145
StbZIP6	PGSC0003DMT400006851	PGSC0003DMG400002660	428	A	343–387
StbZIP7	PGSC0003DMT400007165	PGSC0003DMG402002771	138	S	25–77
StbZIP8	PGSC0003DMT400009068	PGSC0003DMG400003529	144	S	22–69
StbZIP9	PGSC0003DMT400009493	PGSC0003DMG400003701	150	S	27–83
StbZIP10	PGSC0003DMT400009538	PGSC0003DMG401003720	444	I	299–350
StbZIP11	PGSC0003DMT400012575	PGSC0003DMG400004916	140	S	55–107
StbZIP12	PGSC0003DMT400013972	PGSC0003DMG400005476	204	S	86–138
StbZIP13	PGSC0003DMT400015830	PGSC0003DMG401006188	272	F	83–150
StbZIP14	PGSC0003DMT400015892	PGSC0003DMG400006211	414	A	336–381
StbZIP15	PGSC0003DMT400016235	PGSC0003DMG400006345	392	D	60–90
StbZIP16	PGSC0003DMT400016236	PGSC0003DMG400006345	316	D	69–99
StbZIP17	PGSC0003DMT400016237	PGSC0003DMG400006345	355	D	69–99
StbZIP18	PGSC0003DMT400016238	PGSC0003DMG400006345	355	D	69–99
StbZIP19	PGSC0003DMT400018306	PGSC0003DMG400007110	488	D	181–258
StbZIP20	PGSC0003DMT400018486	PGSC0003DMG400007175	822	B	340–398
StbZIP21	PGSC0003DMT400018574	PGSC0003DMG400007208	319	A	249–292
StbZIP22	PGSC0003DMT400018832	PGSC0003DMG400007301	144	S	24–74
StbZIP23	PGSC0003DMT400019465	PGSC0003DMG400007523	194	S	80–138
StbZIP24	PGSC0003DMT400020206	PGSC0003DMG401007810	305	E	179–223
StbZIP25	PGSC0003DMT400020670	PGSC0003DMG400008011	453	A	375–427
StbZIP26	PGSC0003DMT400020671	PGSC0003DMG400008011	448	A	375–425
StbZIP27	PGSC0003DMT400024289	PGSC0003DMG400009388	303	E	175–218
StbZIP28	PGSC0003DMT400024427	PGSC0003DMG400009449	324	D	38–81
StbZIP29	PGSC0003DMT400024428	PGSC0003DMG400009449	324	D	38–81
StbZIP30	PGSC0003DMT400029743	PGSC0003DMG400011430	380	D	83–162
StbZIP31	PGSC0003DMT400030585	PGSC0003DMG400011712	398	A	118–169
StbZIP32	PGSC0003DMT400032381	PGSC0003DMG400012436	289	E	169–213
StbZIP33	PGSC0003DMT400032382	PGSC0003DMG400012436	247	E	169–213
StbZIP34	PGSC0003DMT400032960	PGSC0003DMG400012660	400	G	304–342
StbZIP35	PGSC0003DMT400032965	PGSC0003DMG400012660	351	G	253–315
StbZIP36	PGSC0003DMT400032966	PGSC0003DMG400012660	351	G	253–315
StbZIP37	PGSC0003DMT400033308	PGSC0003DMG400012792	356	D	71–107
StbZIP38	PGSC0003DMT400033741	PGSC0003DMG400012962	483	D	195–236
StbZIP39	PGSC0003DMT400038492	PGSC0003DMG400014858	210	C	37–91
StbZIP40	PGSC0003DMT400038493	PGSC0003DMG400014858	349	C	176–230
StbZIP41	PGSC0003DMT400040054	PGSC0003DMG400015494	132	S	22–80
StbZIP42	PGSC0003DMT400042946	PGSC0003DMG400016651	324	I	133–182
StbZIP43	PGSC0003DMT400043542	PGSC0003DMG400016906	159	S	32–72
StbZIP44	PGSC0003DMT400043648	PGSC0003DMG400016951	246	D	78–117
StbZIP45	PGSC0003DMT400043649	PGSC0003DMG400016951	361	D	78–117
StbZIP46	PGSC0003DMT400043650	PGSC0003DMG400016951	361	D	78–117
StbZIP47	PGSC0003DMT400043651	PGSC0003DMG400016951	361	D	78–117
StbZIP48	PGSC0003DMT400045314	PGSC0003DMG400017577	326	I	171–229
StbZIP49	PGSC0003DMT400047522	PGSC0003DMG400018469	385	G	299–361
StbZIP50	PGSC0003DMT400050096	PGSC0003DMG400019455	451	C	246–299
StbZIP51	PGSC0003DMT400053573	PGSC0003DMG401020784	153	H	85–144
StbZIP52	PGSC0003DMT400053575	PGSC0003DMG401020784	158	H	85–145
StbZIP53	PGSC0003DMT400053576	PGSC0003DMG401020784	185	H	85–144
StbZIP54	PGSC0003DMT400055214	PGSC0003DMG400021431	221	S	117–169
StbZIP55	PGSC0003DMT400058187	PGSC0003DMG400022589	166	S	20–67
StbZIP56	PGSC0003DMT400058468	PGSC0003DMG400022706	146	A	78–132
StbZIP57	PGSC0003DMT400058469	PGSC0003DMG400022706	146	A	78–132
StbZIP58	PGSC0003DMT400059038	PGSC0003DMG400022931	343	A	288–335
StbZIP59	PGSC0003DMT400059039	PGSC0003DMG400022931	366	A	288–346
StbZIP60	PGSC0003DMT400059040	PGSC0003DMG400022931	366	A	288–346
StbZIP61	PGSC0003DMT400060864	PGSC0003DMG400023678	406	D	111–145
StbZIP62	PGSC0003DMT400060865	PGSC0003DMG400023678	461	D	163–223
StbZIP63	PGSC0003DMT400060926	PGSC0003DMG402023696	433	D	147–189
StbZIP64	PGSC0003DMT400061401	PGSC0003DMG400023897	215	A	153–215
StbZIP65	PGSC0003DMT400061402	PGSC0003DMG400023897	191	A	157–179
StbZIP66	PGSC0003DMT400061403	PGSC0003DMG400023897	227	A	153–222
StbZIP67	PGSC0003DMT400064170	PGSC0003DMG401024930	148	E	7–64
StbZIP68	PGSC0003DMT400066603	PGSC0003DMG400025889	414	A	336–386
StbZIP69	PGSC0003DMT400070881	PGSC0003DMG400027562	156	D	77–109
StbZIP70	PGSC0003DMT400070882	PGSC0003DMG400027562	245	D	77–109
StbZIP71	PGSC0003DMT400070883	PGSC0003DMG400027562	361	D	77–109
StbZIP72	PGSC0003DMT400070884	PGSC0003DMG400027562	361	D	77–109
StbZIP73	PGSC0003DMT400070885	PGSC0003DMG400027562	245	D	77–109
StbZIP74	PGSC0003DMT400070968	PGSC0003DMG400027599	349	I	150–198
StbZIP75	PGSC0003DMT400072260	PGSC0003DMG400028121	347	A	277–320
StbZIP76	PGSC0003DMT400074878	PGSC0003DMG400029118	261	I	105–163
StbZIP77	PGSC0003DMT400074879	PGSC0003DMG400029118	185	I	105–163
StbZIP78	PGSC0003DMT400076576	PGSC0003DMG400029774	284	A	203–255
StbZIP79	PGSC0003DMT400076577	PGSC0003DMG400029774	259	A	203–255
StbZIP80	PGSC0003DMT400078084	PGSC0003DMG400030371	184	E	51–110
StbZIP81	PGSC0003DMT400079474	PGSC0003DMG400030946	166	S	30–78
StbZIP82	PGSC0003DMT400084203	PGSC0003DMG400033876	413	G	270–332
StbZIP83	PGSC0003DMT400084204	PGSC0003DMG400033876	337	G	270–332
StbZIP84	PGSC0003DMT400084205	PGSC0003DMG400033876	413	G	270–332
StbZIP85	PGSC0003DMT400084206	PGSC0003DMG400033876	380	G	237–299
StbZIP86	PGSC0003DMT400085559	PGSC0003DMG400035130	138	S	25–77
StbZIP87	PGSC0003DMT400088166	PGSC0003DMG400037737	144	S	23–79
StbZIP88	PGSC0003DMT400089985	PGSC0003DMG400039556	175	E	50–110
StbZIP89	PGSC0003DMT400090249	PGSC0003DMG400039820	671	B	208–265
StbZIP90	PGSC0003DMT400094588	PGSC0003DMG400044159	519	B	179–221

### Phylogenetic analysis of potato bZIPs

**Fig 3:**
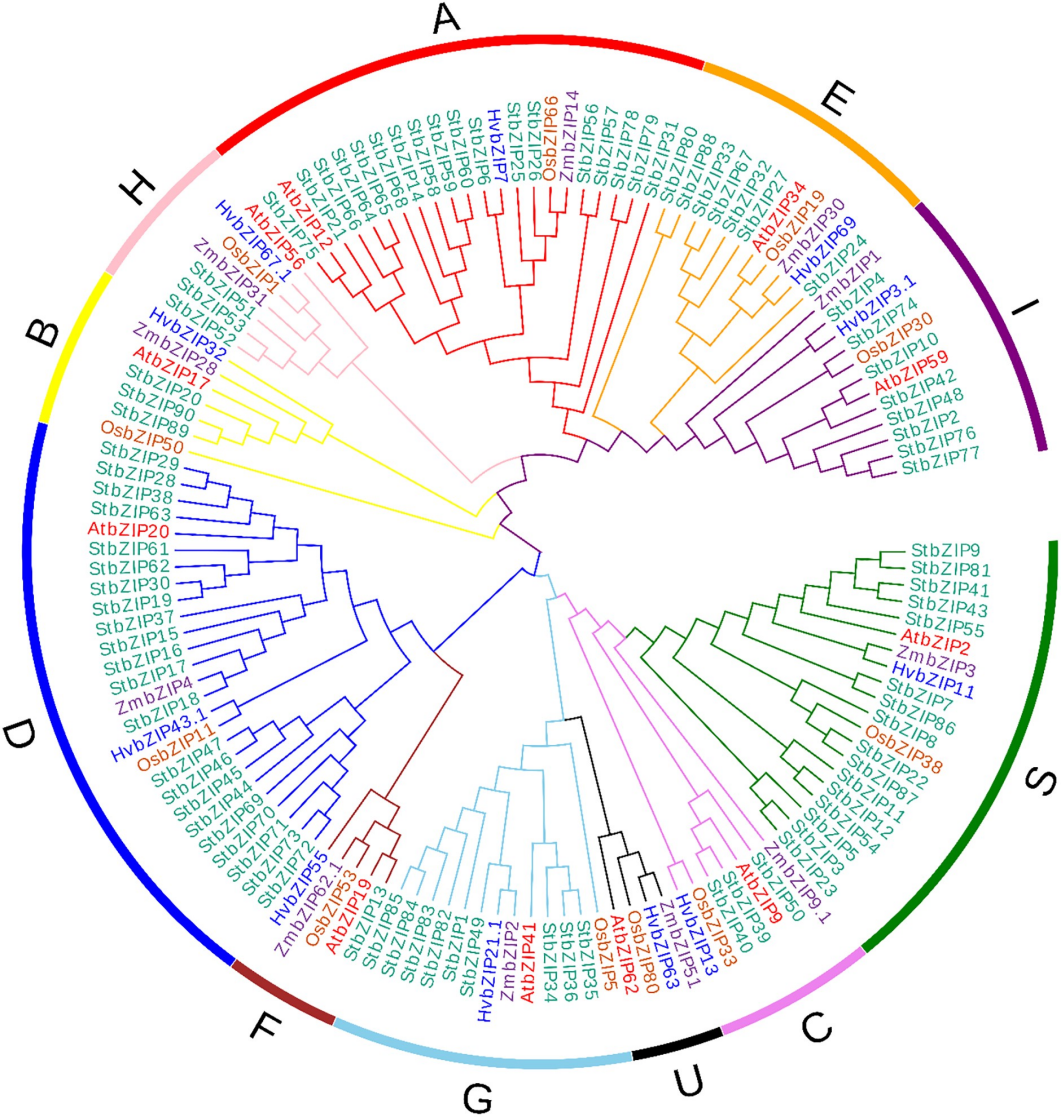
Phylogenetic tree of StbZIPs. The phylogenetic tree with representative bZIPs of each group from *Arabidopsis*, rice, maize, barley, and potato (denoted using red, blue, purple, brown, and green stars, respectively) was constructed using the Neighbor-joining (NJ) method by MEGA 7 with 1000 bootstrap values. The StbZIPs were categorized into 11 groups and are shown by colored branches A to I, S, and U.

The ninety bZIP sequences from potato and representative bZIP sequences from each group were analyzed from rice (*Oryza sativa*), barley (*Hordeum vulgare*), maize (*Zea mays*), and *Arabidopsis* (*Arabidopsis thaliana*). Multiple sequence alignment of StbZIPs with rice, barley, maize, and *Arabidopsis* bZIPs was performed with ClustalW (with gap open penalty 10 and gap extension penalty 0.05) and is represented in S1 Fig. The Neighbor-Joining (NJ) phylogenetic tree was constructed using MEGA7.0 and visualized by EvolView (Fig 3). The phylogenetic tree analysis grouped StbZIPs proteins into 11 clades, designated as groups A to I, S, and U, following those in *Arabidopsis* [10]. Among these, groups A and D constituted the largest clades with 11 and 22 members, respectively. StbZIP members were placed within category C and B (3 StbZIPs each), group I (8 StbZIPs), group G (9 StbZIPs), group S (16 StbZIPs), group E (7 StbZIPs), group F (1 StbZIP), and group H (3 StbZIPs) (Figs 2 and 3). Group A is one of the major groups comprising 11 StbZIPs, (*StbZIP*s 3, 14, 15, 16, 17, 18, 19, 20, 21, 22). It is evident from prior studies that group A StbZIPs have an important role in abscisic acid (ABA) signaling and tolerance to abiotic stresses as evident by transcriptional and post-translational regulation of its group members [10, 39]. Recently, in rice, *OsbZIP23* was reported to have a significant role in tolerance to salinity and drought (ABA-dependent) [40]. Group D is composed of 22 *StbZIPs*, one of the largest potato groups. This group’s genes are primarily involved in plant development and defense-related pathways [10, 14]. Earlier findings on the bZIP domain containing TGA family (TGACG sequence binding protein) in *Arabidopsis* and tobacco reported a pivotal role in stress defense mechanisms [41, 42]. The TGA family generally interacts with the Non-Expressor of PR1 (NPR1) proteins, so this protein is a central component in the defense signaling related to salicylic acid [43, 44]. The *Arabidopsis* TGA2 protein interacts with the xenobiotic responsive promoters that trigger the reaction process linked to salicylic acid pathways. A similar form of the process has been found in tobacco, as pathways are activated by interaction with TGA1a [14, 41, 44]. Group B and C are among the smallest groups in the potato bZIP family that consists of three StbZIPs. The function-related information of group B is limited. The earlier published reports showed that Group B bZIPs have a transmembrane domain with specific stretches of amino acids at the C-terminus and play significant roles in salt stress response via endoplasmic reticulum stress signaling [10]. The members of Group C bZIPs have an essential role in modulating seed-specific gene expression [10, 14]. The rice *OsbZIP52* from group C showed a significant increase in the susceptivity to drought and cold stresses during the seed germination process [45].

Group E and F are constituted by seven and one bZIP members, respectively. The members of these groups, e.g. bZIP 34 and 61, are reported to have a role in the pollen germination and tube growth in *Arabidopsis* [46]. Group F is primarily responsible for regulating genes involved in zinc transport, thus promoting plant adaptation through binding to zinc-deficiency-response elements (ZDREs; RTGTCGACAY) under zinc-starving conditions [47]. Group G consists of nine StbZIPs. The name assigned by the letter ‘G’ refers to G-box, which also function as binding factors for bZIPs [48]. The G-box binding factor bZIPs in *Arabidopsis* are attributed to the regulation of light-responsive elements and perform a function in cell protection during both biotic and abiotic stress [49, 50]. Group H is comprised of three StbZIPs. For several plant species possessing a complete conserved pattern of the bZIP domain, this group’s name is often known as the *HY5* gene. In *Arabidopsis*, the gene *HY5* attaches to the G-box and regulates root and hypocotyl development [51]. Potato Group I consists of eight bZIPs, and all members of the group I have a lysine (K) instead of arginine (R) at the 10^th^ position [10, 14]. Members of group I bZIPs are reported to be functionally involved in vascular development [10, 11, 12]. Group S bZIPs in potato is the second largest group, with sixteen bZIPs, although it is the main group in *Arabidopsis*. The small size of proteins in this group specifies their requirement to interact with certain other transcriptional activation factors [10, 52]. The bZIP proteins from *Arabidopsis* (*AtbZIP1*), *Medicago* (*MtbZIP2*, and *MtbZIP26*), and rice (*OsbZIP16*) showed higher expression during salt treatment, resulting in an improvement in salt stress tolerance in several crops [53]. Group U in potato consisted of five StbZIP members which share high sequence similarity to the same members from other crops, including maize and rice. Conserved domains for members of this group have a set of signature sequences in the hinge and basic regions, and a conserved amino acid residue isoleucine is replaced with arginine. This substitution affects the DNA binding specificity as reported in earlier studies, and such a substitution has shown to inhibit the affinity of bZIP for AP1 protein in yeast [10] and rice for promoters containing G-boxes [11].

### StbZIP properties and conserved motif analysis

Analysis of structure and divergence using the MEME tool led to the identification of a total of 10 motifs in all the 90 StbZIPs (S1 Table, Fig 2). The basic leucine zipper domain of motif 1 is commonly present in all the StbZIP proteins. Besides group D, motif 1 to 6 is present in all the groups. Groups A, B, C, F, G, and I share motifs 1, 7, 8, and 9. Present results show that StbZIPs share similar sequences and are clustered in the same group. Prior studies reported that monocots usually have more bZIP and motifs than the others [54]. In wheat, a total of 370 bZIPs were reported and categorized into 11 groups (A to I, S, and U) [11]. Similarly, in potato, these bZIPs were grouped into ten groups (lacking less members in group U) based on functional annotation (Fig 3). Even the same conserved pattern was observed in the MEME motif pattern in all the groups (Fig 2). The StbZIP proteins from a similar group had the same motifs arranged in the same order (S1 Table).

### Genome-wide expression atlas of *StbZIP*s

Using FPKM data, tissue-specific expression dynamics of *StbZIPs* were studied. The effect of various abiotic stresses and hormones on the expression of *StbZIPs* was also analyzed.

#### Tissue-specific expression dynamics (leaf, root, tuber, sepal, petal, mesocarp & endocarp)

The leaf-stage expression study showed an expression of a total of forty-seven *StbZIPs*. Out of these, thirty-four exhibited an expression value of >2 FPKM. In roots, fifty-five *StbZIPs* were expressed, and forty of them had the FPKM value of >2. Out of these, thirty-six *StbZIPs* had expression >5 FPKM, and *StbZIP* 25, 34, 50, and 89 had a maximum expression. Forty-eight *StbZIPs* were expressed in tubers, and *StbZIP* 4, 22, 34, 42, 50, 63, 68, and 89 had the highest expression. In the sepal and petal stage, fifty-one and fifty-two *StbZIP* were expressed, and out of these six *StbZIPs*, each showed maximum expression in sepal (*StbZIP* 20, 25, 34, 50, 63, and 89) and petal (StbZIP 20, 25, 34, 75, 87 and 89). During the mesocarp & endocarp development (fruiting stage), expression analysis revealed fifty-four and forty-four *StbZIPs* with expression > 2 FPKM. However, only thirty-eight *StbZIPs* showed > 5 FPKM with the highest expression in *StbZIP* 20, 34, 42, 50, 87, and 89. Expression analysis based on FPKM suggested that most *bZIPs* are expressed in all tissues. *StbZIP 25* had a higher expression in root, sepal, and petals, and *StbZIP* 89 and 34 had the highest expression in all tissues, i.e., root, tuber, sepal, petal, and fruit. Similarly, *StbZIP* 50 showed the highest expression in root, tuber, sepal, and fruit. *StbZIP* 42 showed the common highest expression in tuber and fruit (S2 Table). The expression analysis suggested the tissue-specific expression of a few, and the ubiquitous expression of most, *StbZIPs*. This expression atlas can further be used to identify candidate *StbZIPs* for crop improvement by developing cis/trans-genic lines. Using a similar approach, *CabZIP25* was identified from *Capsicum annum* and its role in imparting salt stress tolerance was shown by heterologous expression in *Arabidopsis* [54].

#### Abiotic stress-based expression dynamics (salinity, osmotic, water, and heat)

**Fig 4:**
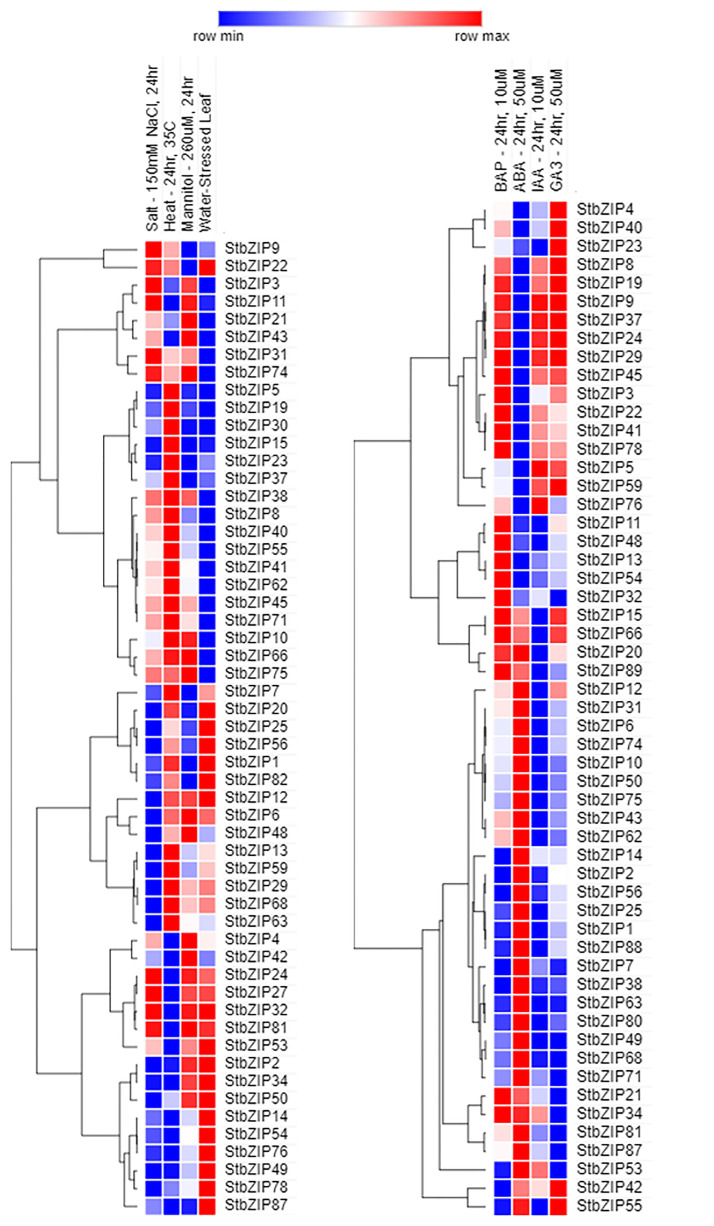
Differential expression (log 2 FC) of *StbZIPs* under various abiotic stresses. A heat map represents the log 2 FC-FPKM value of *StbZIPs* after stress induced by NaCl, mannitol, heat, water, and exogenous application of phytohormones BAP, ABA, IAA, and GA3.

Abiotic stress, such as salt, mannitol, water, and heat, can significantly impact the production of the potato crop. Salinity affects plant development and growth through interference with the plant’s physiological system. The FPKM expression values were used to calculate the fold change (FC) and log 2 FC by comparing controls with respective treated samples (Fig 4, S3 Table). The comparative analysis showed fifty-five *StbZIPs* in leaf tissues after treatment with 150 mM NaCl. Out of these, thirteen had an expression of > 2 FC, and thirty-one of *<* 2 FC. However, differential log 2 FC expression analysis showed that only *StbZIP* 62 and 66 showed positive differential log 2 FC in salt stress condition. *StbZIPs* that showed negatively differential log 2 FC expression during this salt stress were *StbZIP* 7, 2, 4, 6, 23, 32, 38, 48, 49, 75, and 71. These *bZIPs* could serve as negative regulators during salt stress.

Fifty-five *StbZIPs* were expressed under mannitol stress (concentration of 260 μM for 24 h), out of which only 17 had an expression value of > 2FC. Among these, S*tbZIPs* 21and 66 showed expression of FC >5. Forty-eight *StbZIPs* were expressed during water stress, out of which six *StbZIPs* had an FC >5. Among these, *StbZIP*1, 14, 25, 53, 54 and 82 had log2 FC values of >2. Fifteen *StbZIPs* were negatively regulated under water stress, and only *StbZIP*3, 38, 62, and 71 had negative log2FC >2. Based on this data, *StbZIPs* can be further selected for functional validation using forward and reverse genetics approaches.

Salinity stress can cause osmotic imbalance and toxicity to ions. The major impact of osmotic stress on plants is on growth reduction due to reduced water potential [54]. The salt stress also increases sodium ion (Na^+^) concentration that directly hinders the absorption of potassium ions (K^+^) in plants. However, in higher plants, low Na^+^ levels, and a normal range of K^+^/Na^+^ ratios enhance salt tolerance in the cell cytoplasm [55, 56]. The exact mechanism of tolerance to salt and osmotic stress in potato is not well studied, especially how *bZIPs* can regulate this response. In the present study, initial evidence of expression of *StbZIPs* during abiotic stress conditions sets out a new hypothesis to understand the regulatory process as validated in prior reports, which showed that expression of *bZIP17* increased salt tolerance in *Arabidopsis* [57]. Similarly, the *AREB1* and *ABF2* are *bZIP* family genes, and their overexpression and silencing in tomato and cotton resulted in enhanced salt- and drought-stress tolerance, respectively [58, 59]. The *AREB1* gene also regulates other genes related to abiotic and biotic stress [58–60].

The process of tuberization is largely dependent on photoperiod [61], phytohormones [62], and day and night temperature [63]. The exact mechanism of potato tuberization and the effect of temperature on potatoes is not well understood. However, heat stress can lead to reduced yield due to less photosynthate available for plant growth and partitioning into potato tubers [64]. Heat stress causes physiological changes in tubers like early sprouting, altered tuber shape, tuber cracking, changes in texture, taste formation, low dry matter, etc. Also, heat stress can induce necrosis, loss of chlorophyll with reduced stem thickness, and an increase in the quantity of reducing sugars [64]. Henceforth, a detailed understanding of the regulatory network is needed to develop lines that provide resistance/tolerance to heat stress. In the present study, we identified fifty-four *StbZIPs* expressed during heat stress (heating at 35 °C for 24h), and out of these, a total of twelve *StbZIPs* had FC >5. The *StbZIPs* 1, 5, 7, 8, 15, 19, 23, 30, 37, 41, 62, 66 and 82 had positive expression of > 2 FC, whereas *StbZIP* 3, 24, 27, 32, and 53 were negatively expressed. The overall expression study of *StbZIPs* during abiotic (heat, water, salt, mannitol) stress conditions led to the identification of a total of 33 *StbZIPs* stresses (Fig 4, S3 and S4 Tables) that can be targeted to improve resistance/tolerance against abiotic stresses.

### Hormone-induced expression profiles of *StbZIPs*

Phytohormones are the critical players in inducing cell divisions, and partitioning assimilates by source-sink interactions [65] and are active in the transcriptional regulation of various stress-related genes [66]. Therefore, the expression of *StbZIPs* was studied after treatment with different hormones (Fig 4, S3 and S5 Tables).

#### BAP (10 μM 24h)

The exogenous application of BAP phytohormones resulted in the expression of a total of 55 *StbZIPs*, but none of them revealed the expression of more than 2 FPKM. Differential log2 FC FPKM expression analysis suggested the downregulation of most of the *StbZIPs*.

#### ABA (50 μM 24h)

The expression analysis after the exogenous supply of ABA in potato leaf tissue revealed 53 *StbZIPs*, and out of these, only *StbZIP88* had an expression of >2 FC. However, relative differential log2 FC expression analysis suggests that four StbZIPs are positively (1, 7, 25, and 88) and negatively (8, 21, 24, and 37) expressed, respectively. ABA plays a vital role in plant physiological processes and, therefore, regulates plant development. The expression of several ABA/stress-responsive genes in plants is regulated by cis-acting elements [66, 67]. The regulation of ABA is reported to be controlled by PYRABACTIN RESISTANCE (PYR / PYL), PROTEIN PHOSPHATASE 2C, SNF1-RELATED PROTEIN KINASE 2 (SnRK2), ion channels, and NADPH oxidases [68], and most notably by bZIPs. The bZIP has a binding affinity towards the ABA-responsive element-binding protein / ABRE-binding factor (AREB / ABF) [69]. The identified ABA-responsive *StbZIPs* will facilitate further functional characterization of these subfamily members.

#### IAA (10 μM 24h)

Auxin, particularly indole-3-acetic acid (IAA), is a plant growth hormone that affects plants’ growth and development [70]. Following treatment of 10 mM IAA, an analysis of the differential log 2-fold expression showed that only three *StbZIPs* (5, 32, and 59) exhibit positive regulation, while a set of 18 *bZIPs* are down-regulated. IAA regulates various biological and physiological processes such as cell elongation, induction of root growth, and flower and fruit development [71]. Such responses are controlled by the cis-acting auxin-responsive elements (AuxREs) [70], which are directly linked by auxin response factors (ARFs) [70, 71] by protein-protein interaction and repressed by AUX / IAA protein transcription [72]. The promoter analysis in prior studies suggests that other cis-elements are also involved in auxin-mediated transcription in addition to AuxREs. For example, the soybean and tobacco GH3 promoters found a conserved organization of G-box-related elements (GREs) located near AuxREs [72]. An enrichment of modules consisting of GREs and AuxREs in auxin-inducible promoters in *Arabidopsis* and rice was reported using *in silico* bioinformatics approaches [72, 73]. Based on our expression data, and available literature, it is evident that *bZIPs* regulate ABA / IAA stress in potato.

#### GA3 (50 μM 24h)

Gibberellins are a large family of plant growth hormones. The gibberellic acid (GA3) is mainly involved in cell expansion, cell division, seed germination, and stem elongation. GA3 performs a versatile function in the growth and production of Potatoes, and, at a low concentration, it induces the seed tuber weight and sugar content [74]. A total of five *StbZIPs* (8, 23, 32, 59, and 88) showed expression greater than two-fold (FC) upon treatment with GA3.

### Development of SSR markers

Mining existing diversity and functional validation in a large population are vital for germplasm conservation and crop improvement. bZIPs play crucial roles in plants in stress and developmental processes. Therefore, it is important to develop molecular markers that can be used in breeding programs [75, 76]. Out of 30 candidate *StbZIPs*, only sixteen had SSR containing sequences from which 22 SSRs were identified (Table 2). The highest number of tri repeats was eleven among these SSRs. Detailed information regarding SSR-like motif frequency and frequency of classified repeat types are available in Table 2 and S6 Table. It is important to note that these SSR markers were derived from *StbZIPs* which were differentially regulated in a stress-specific manner. For example, *StbZIP*2, 4, 7, 23, 38, 71, 75 were differentially regulated under salt stress, *StbZIP*25, 54, and 82 under mannitol stress, *StbZIP38* under water stress, *StbZIP*7 and 25 under ABA stress, *StbZIP*5 under IAA stress, and *StbZIP*23 under GA3 stress (S6 Table). These candidate *StbZIPs* and SSRs add to the list of known genes and markers and after validation can be further used in the marker-trait analysis to breed potato for tolerance to abiotic stresses. A similar approach was adopted to identify candidate *bZIPs* and develop SSR markers in *Medicago truncatula* [75] and wheat [76].

**Table 2:** Mining of SSRs from candidate *StbZIP*s.

The abundance of candidate bZIP SSR in potato (cDNA sequences)	Distribution to different repeat type		Frequency of identified SSR motifs							Frequency of classified repeat types (considering sequence complementary)						
Total number of sequences examined (30)	Unit size	Number of SSRs	Repeats	3	4	5	6	7	Total	Repeats	3	4	5	6	7	Total
Total size of examined sequences (bp) **(44119)**	2	1	AG				1		1	AG/CT				1		1
Total number of identified SSRs **(22)**	3	11	AAC		1				1	AAC/GTT		3			1	4
Number of SSR containing sequences **(16)**	4	6	ATC					1	1	AAG/CTT		1				1
Number of sequences containing more than one SSR **(3)**	5	2	CAA		2			1	3	AAT/ATT		1				1
Number of SSRs present in compound formation **(1)**	6	2	CAG		1				1	ACC/GGT				1	1	2
			CCT		1				1	AGC/CTG		1				1
			GAA		1				1	AGG/CCT		1				1
			GGT				1		1	ATC/ATG					1	1
			GTG					1	1	AAAC/GTTT	2					2
			TAA		1				1	AAAG/CTTT	2					2
			AAAG	1					1	AAGG/CCTT	1					1
			CATT	1					1	AATG/ATTC	1					1
			CTTT	1					1	AAGGT/ACCTT	1					1
			TCCT	1					1	AATAC/ATTGT	1					1
			TGTT	2					2	AAAATC/ATTTTG			1			1
			ACCTT	1					1	AAGCTC/AGCTTG	1					1
			ATACA	1					1							
			CTCAAG	1					1							
			TGATTT			1			1							

## Conclusions

In summary, a total of 90 *StbZIPs* on a genome-wide level were identified in potato. The FPKM based expression analysis led to the identification of eighteen candidate *StbZIPs*, expressed in a tissue-specific manner [4 in roots (*StbZIP* 25, 34, 50, and 89), 8 in tubers (*StbZIP* 4, 22, 34, 42, 50, 63, 68, and 89), and four each in mesocarp and endocarp (*StbZIP* 20, 34, 42, 50, 87, and 89)]. Differential expression analysis (log 2 FC) under salt stress identified 2 positively (*StbZIP* 62 and 66), and 11 negatively (*StbZIPs* 7, 2, 4, 6, 23, 32, 38, 48, 49, 71, and 75) dysregulated *StbZIPs*. In response to water stress, *StbZIPs* 1, 14, 25, 53, 54, 82 showed positive upregulation (log2FC), while *StbZIPs* 3, 38, 62, and 71 were regulated negatively. The application of mannitol on potato showed the upregulated of *StbZIPs* 21 and 66. Heat stress led to positive up-regulation of *StbZIPs* 1, 5, 7, 8, 15, 17, 23, 30, 37, 41, 62, 66, and 82, while StbZIPs 3, 24, 27, 32, 53 were upregulated but negatively. Likewise, the expression analysis upon treatment with phytohormones led to the identification of 8 *StbZIP* that were dysregulated upon ABA treatment [*StbZIP* 1, 7, 25, and 88 were positively upregulated, and *StbZIPs* 8, 21, 24, and 37 were negative downregulated], and three upon IAA treatment (*StbZIPs* 5, 32, and 59 upregulated positively). No major changes in the expression of *StbZIPs* were observed upon treatment with IAA and BAP. The phylogenetic analysis, annotation of function, and expression analysis opens the path for further functional validation studies and the use of *StbZIPs* for engineering potato for tolerance to various abiotic stresses. Similarly, the identified SSRs from candidate *StbZIPs* will also be useful in potato crop improvement programs. The function of these genes can be validated by using either overexpression or gene knockout approaches.

## Supporting information

S1 Fig Multiple sequence alignment of basic and hinge regions of *StbZIPs*. The StbZIP proteins were classified into 11 categories (A-I, S, and U). Each group’s representative bZIP was taken as reference from *Arabidopsis*, rice, maize, and barley and is shown in the red box. An asterisk marks conserved amino acid residues.
(PDF)

S1 Table Motif analysis of StbZIPs with the group name, length, motif type, start, and end position. (XLSX)

S2 Table FPKM expression of *StbZIPs* in different tissues (leaves, roots, tuber, sepals, petals, and fruit). The FPKM values were retrieved from the various transcriptomic datasets available at http://solanaceae.plantbiology.msu.edu/pgsc_download.shtml.
(DOCX)

S3 Table FPKM expression during control and abiotic stress conditions were retrieved from http://solanaceae.plantbiology.msu.edu/pgsc_download.shtml in potato tissues. (XLSX)

S4 Table Differential log 2 FC- FPKM, expression of StbZIPs in abiotic conditions (salt-150 mM NaCl for 24h, heat-35 °C for 24h, mannitol-260 μM for 24h, and water stress) and the controls in potato plants at different times and concentrations. (DOCX)

S5 Table Differential log 2 FC-FPKM expression analysis after the exogenous supply of phytohormones BAP, ABA, IAA, and GA for control in potato plant at different concentrations and times. (DOCX)

S6 Table List and primer sequence information of identified SSR of candidate StbZIPs. (DOCX)
