# Supplementary material for: Genome-wide identification and expression profiling of basic leucine zipper transcription factors following abiotic stresses in potato (Solanum tuberosum L.)
Source: PLoS One. 2021 Mar 12;16(3):e0247864. doi: 10.1371/journal.pone.0247864 (PMC7954325; doi:10.1371/journal.pone.0247864)
Supplement: S1 Fig — The StbZIP proteins were classified into 11 categories (A-I, S, and U). Each group’s representative bZIP was taken as reference from Arabidopsis, rice, maize, and barley and is shown in the red box. An asterisk marks conserved amino acid residues. (PDF) [file pone.0247864.s001.pdf]

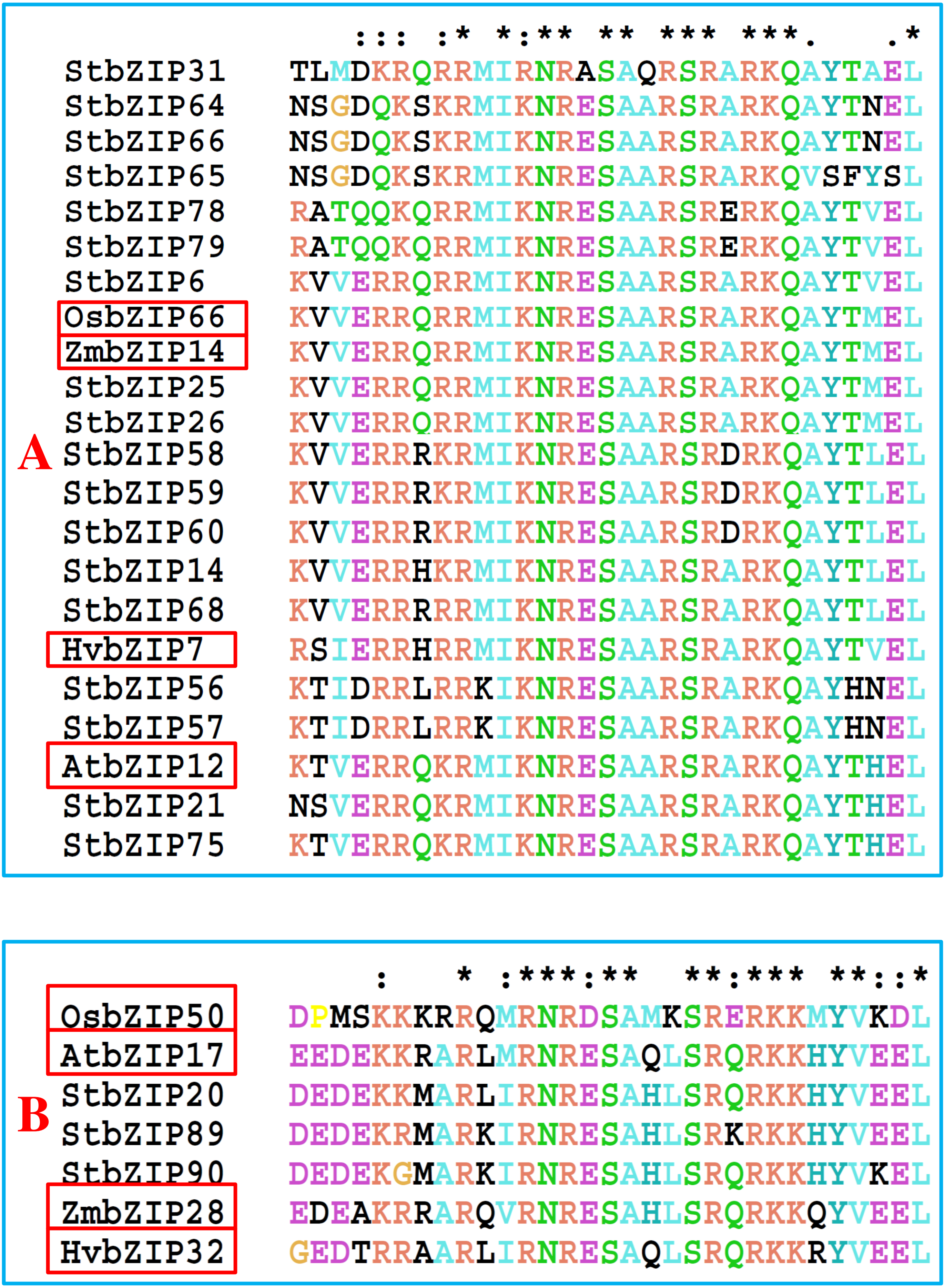

A
::: :* *:** ** *** ***. .*
StbZIP31 TLMDKRQRRMIRNRASAQRSRARKQAYTAEL
StbZIP64 NSGDQKSKRMIKNRESAARSRARKQAYTNEL
StbZIP66 NSGDQKSKRMIKNRESAARSRARKQAYTNEL
StbZIP65 NSGDQKSKRMIKNRESAARSRARKQVSFYSL
StbZIP78 RATQQKQRRMIKNRESAARSRERKQAYTVEL
StbZIP79 RATQQKQRRMIKNRESAARSRERKQAYTVEL
StbZIP6 KVVERRQRRMIKNRESAARSRARKQAYTVEL
OsbZIP66 KVVERRQRRMIKNRESAARSRARKQAYTMEL
ZmbZIP14 KVVERRQRRMIKNRESAARSRARKQAYTMEL
StbZIP25 KVVERRQRRMIKNRESAARSRARKQAYTMEL
StbZIP26 KVVERRQRRMIKNRESAARSRARKQAYTMEL
StbZIP58 KVVERRRKRMIKNRESAARSRDRKQAYTLEL
StbZIP59 KVVERRRKRMIKNRESAARSRDRKQAYTLEL
StbZIP60 KVVERRRKRMIKNRESAARSRDRKQAYTLEL
StbZIP14 KVVERRHKRMIKNRESAARSRARKQAYTLEL
StbZIP68 KVVERRRRRMIKNRESAARSRARKQAYTLEL
HvbZIP7 RSIERRHRRMIKNRESAARSRARKQAYTVEL
StbZIP56 KTIDRRLRRKIKNRESAARSRARKQAYHNEL
StbZIP57 KTIDRRLRRKIKNRESAARSRARKQAYHNEL
AtbZIP12 KTVERRQKRMIKNRESAARSRARKQAYTHEL
StbZIP21 NSVERRQKRMIKNRESAARSRARKQAYTHEL
StbZIP75 KTVERRQKRMIKNRESAARSRARKQAYTHEL
B
: * :***:** **:*** **::*
OsbZIP50 DPMSKKKRRQMRNRDSAMKSRERKKMYVKDL
AtbZIP17 EEDEKKRARLMRNRESAQLSRQRKKHYVEEL
StbZIP20 DEDEKKMARLIRNRESAHLSRQRKKHYVEEL
StbZIP89 DEDEKRMARKIRNRESAHLSRKRKKHYVEEL
StbZIP90 DEDEKGMARKIRNRESAHLSRQRKKHYVKEL
ZmbZIP28 EDEAKRRARQVRNRESAHLSRQRKKQYVEEL
HvbZIP32 GEDTRRAARLIRNRESAQLSRQRKKRYVEEL

C

```
            * : :  **  ******:*** **  :* :*
AtbZIP9     PNDLKRIRRMNSNRESAKRSRRRKQEYLVDL
StbZIP39    PSDIKRIRRQASNRESARRSRRRKQAHLADL
StbZIP40    PSDIKRIRRQASNRESARRSRRRKQAHLADL
ZmbZIP9.1   PANAKKMRRMVSNRESARRSRKRKQAHLTDL
StbZIP50    PADTKRVRRMLSNRESARRSRRRKQAHLTEL
OsbZIP33    PADQRLQRRKQSNRESARRSRSRKAAHLNEL
HvbZIP13    PTDQRLRRRKQSNRESARRSRSRKAAHLNEL
```

D

```
                *. **********:***:*****:*:*
StbZIP44    SVSDKVQRRLAQNREAARKSRMRKKAYVQQL
StbZIP45    SVSDKVQRRLAQNREAARKSRMRKKAYVQQL
StbZIP46    SVSDKVQRRLAQNREAARKSRMRKKAYVQQL
StbZIP47    SVSDKVQRRLAQNREAARKSRMRKKAYVQQL
StbZIP69    SSSDKAQRRLAQNREAARKSRMRKKAYVQQL
StbZIP70    SSSDKAQRRLAQNREAARKSRMRKKAYVQQL
StbZIP73    SSSDKAQRRLAQNREAARKSRMRKKAYVQQL
StbZIP71    SSSDKAQRRLAQNREAARKSRMRKKAYVQQL
StbZIP72    SSSDKAQRRLAQNREAARKSRMRKKAYVQQL
StbZIP37    KRIDKVRRRLAQNREAARKSRLRKKAYVQQL
StbZIP16    KPIEKVLRRLAQNREAARKSRLRKKAYVQQL
StbZIP15    KPIEKVLRRLAQNREAARKSRLRKKAYVQQL
StbZIP17    KPIEKVLRRLAQNREAARKSRLRKKAYVQQL
StbZIP18    KPIEKVLRRLAQNREAARKSRLRKKAYVQQL
StbZIP30    TTDHKTLRRLAQNREAARKSRIRKKAYIQQL
StbZIP61    ----KTLRRLAQNREAARKSRIRKKAYVQQL
StbZIP62    TSDPKTLRRLAQNREAARKSRIRKKAYVQQL
OsbZIP11    SVDAKTERRLAQNREAARKSRLRKKAYVQNL
HvbZIP43.1  LVDPKTERRLAQNREAARKSRLRKKAYVQQL
StbZIP19    SNSEKTLRRLAQNREAAKKSRIRKKAYVQQL
StbZIP38    LGDQKTLRRLAQNREAARKSRLRKKAYVQQL
StbZIP63    VLDQKTLRRLAQNREAARKSRLRKKAYVQQL
AtbZIP20    KMDQKTLRRLAQNREAARKSRLRKKAYVQQL
StbZIP28    PKDQKTLRRLAQNREAARKSRLRKKAYVQQL
StbZIP29    PKDQKTLRRLAQNREAARKSRLRKKAYVQQL
ZmbZIP4     PMDQKVLRRLAQNREAARKSRLRKKAYVQQL
```

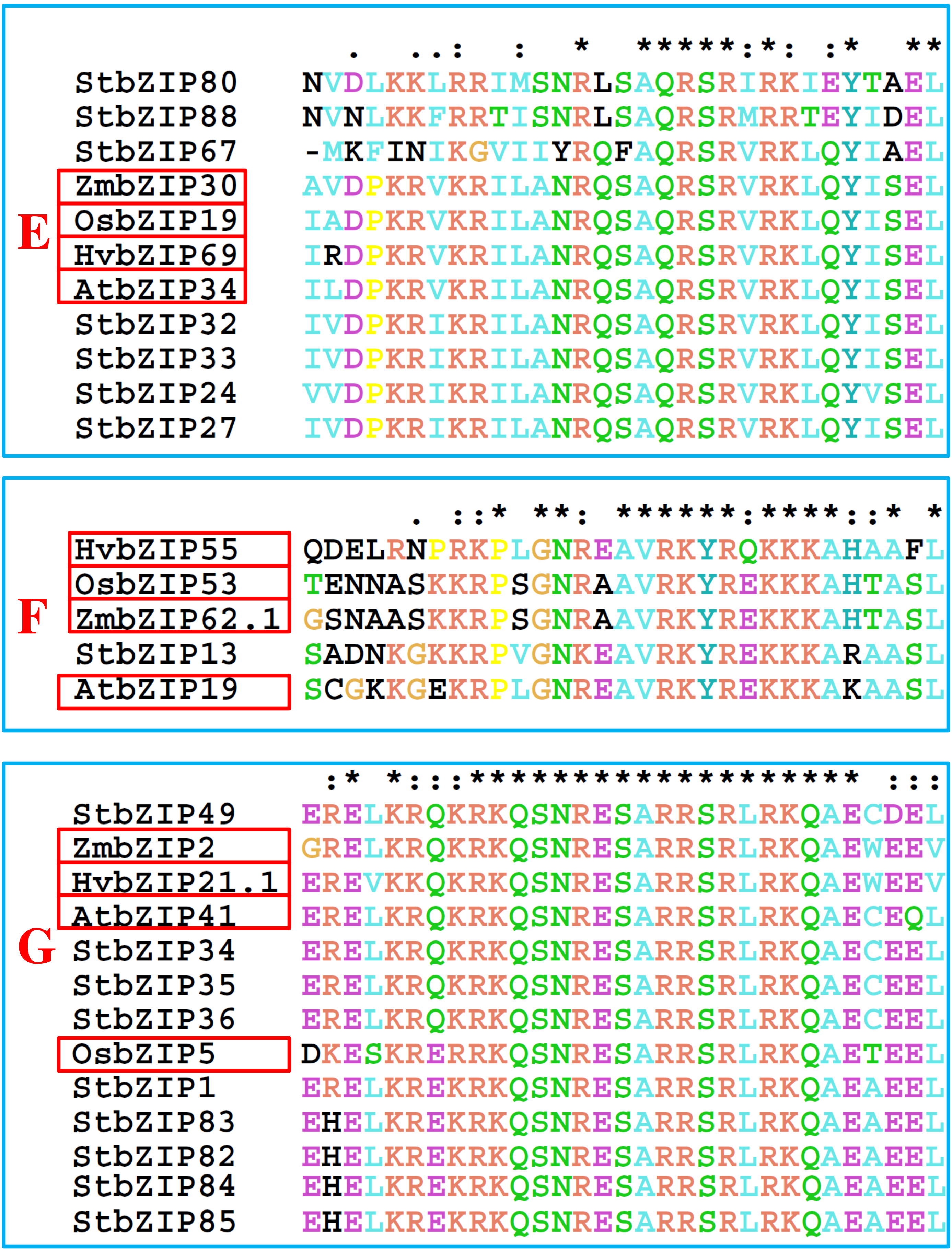
E
. ..: : * *****:*: :* **
StbZIP80 NVDLKKLRRIMSNRLSAQRSRIRKIEYTAEL
StbZIP88 NVNLKKFRRTISNRLSAQRSRMRRTEYIDEL
StbZIP67 -MKFINIKGVIIYRQFAQRSRVRKLQYIAEL
ZmbZIP30 AVDPKRVKRILANRQSAQRSRVRKLQYISEL
OsbZIP19 IADPKRVKRILANRQSAQRSRVRKLQYISEL
HvbZIP69 IRDPKRVKRILANRQSAQRSRVRKLQYISEL
AtbZIP34 ILDPKRVKRILANRQSAQRSRVRKLQYISEL
StbZIP32 IVDPKRIKRILANRQSAQRSRVRKLQYISEL
StbZIP33 IVDPKRIKRILANRQSAQRSRVRKLQYISEL
StbZIP24 VVDPKRIKRILANRQSAQRSRVRKLQYVSEL
StbZIP27 IVDPKRIKRILANRQSAQRSRVRKLQYISEL
F
. ::* **: ******:****::* *
HvbZIP55 QDELRNPRKPLGNREAVRKYRQKKKAHAAFL
OsbZIP53 TENNASKKRPSGNRAAVRKYREKKKAHTASL
ZmbZIP62.1 GSNAASKKRPSGNRAAVRKYREKKKAHTASL
StbZIP13 SADNKGKKRPVGNKEAVRKYREKKKARAASL
AtbZIP19 SCGKKGEKRPLGNREAVRKYREKKKAKAASL
G
:* *:::******************** :::
StbZIP49 ERELKRQKRKQSNRESARRSRLRKQAECDEL
ZmbZIP2 GRELKRQKRKQSNRESARRSRLRKQAEWEEV
HvbZIP21.1 EREVKKQKRKQSNRESARRSRLRKQAEWEEV
AtbZIP41 ERELKRQKRKQSNRESARRSRLRKQAECEQL
StbZIP34 ERELKRQKRKQSNRESARRSRLRKQAECEEL
StbZIP35 ERELKRQKRKQSNRESARRSRLRKQAECEEL
StbZIP36 ERELKRQKRKQSNRESARRSRLRKQAECEEL
OsbZIP5 DKESKRERRKQSNRESARRSRLRKQAETEEL
StbZIP1 ERELKREKRKQSNRESARRSRLRKQAEAEEL
StbZIP83 EHELKREKRKQSNRESARRSRLRKQAEAEEL
StbZIP82 EHELKREKRKQSNRESARRSRLRKQAEAEEL
StbZIP84 EHELKREKRKQSNRESARRSRLRKQAEAEEL
StbZIP85 EHELKREKRKQSNRESARRSRLRKQAEAEEL

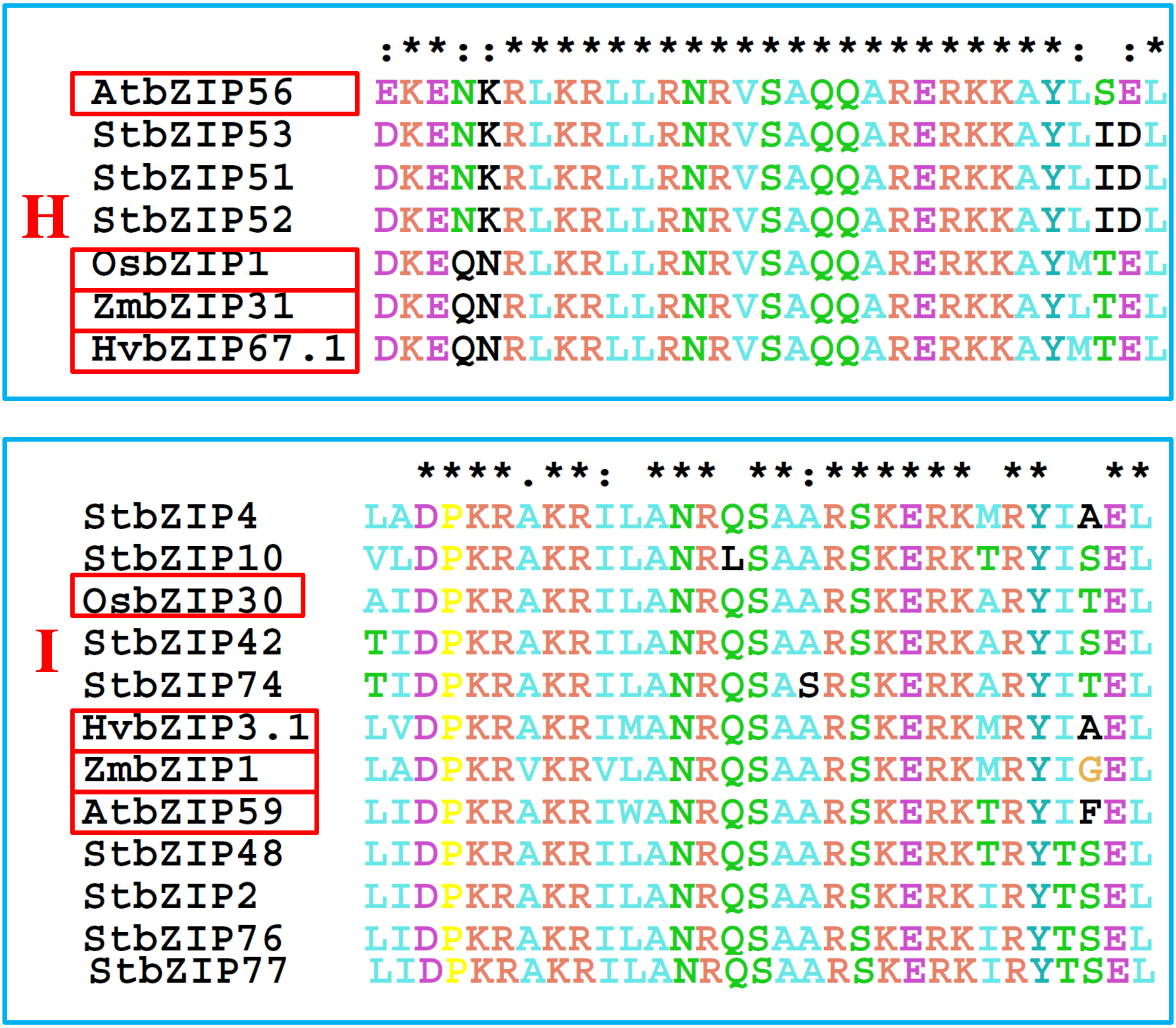
H
:**::**********************: :*
AtbZIP56 EKENKRLKRLLRNRVSAQQARERKKAYLSEL
StbZIP53 DKENKRLKRLLRNRVSAQQARERKKAYLIDL
StbZIP51 DKENKRLKRLLRNRVSAQQARERKKAYLIDL
StbZIP52 DKENKRLKRLLRNRVSAQQARERKKAYLIDL
OsbZIP1 DKEQNRLKRLLRNRVSAQQARERKKAYMTEL
ZmbZIP31 DKEQNRLKRLLRNRVSAQQARERKKAYLTEL
HvbZIP67.1 DKEQNRLKRLLRNRVSAQQARERKKAYMTEL
I
****.**: *** **:****** ** **
StbZIP4 LADPKRAKRILANRQSAARSKERKMRYIAEL
StbZIP10 VLDPKRAKRILANRLSAARSKERKTRYISEL
OsbZIP30 AIDPKRAKRILANRQSAARSKERKARYITEL
StbZIP42 TIDPKRAKRILANRQSAARSKERKARYISEL
StbZIP74 TIDPKRAKRILANRQSASRSKERKARYITEL
HvbZIP3.1 LVDPKRAKRIMANRQSAARSKERKMRYIAEL
ZmbZIP1 LADPKRVKRVLANRQSAARSKERKMRYIGEL
AtbZIP59 LIDPKRAKRIWANRQSAARSKERKTRYIFEL
StbZIP48 LIDPKRAKRILANRQSAARSKERKTRYTSEL
StbZIP2 LIDPKRAKRILANRQSAARSKERKIRYTSEL
StbZIP76 LIDPKRAKRILANRQSAARSKERKIRYTSEL
StbZIP77 LIDPKRAKRILANRQSAARSKERKIRYTSEL

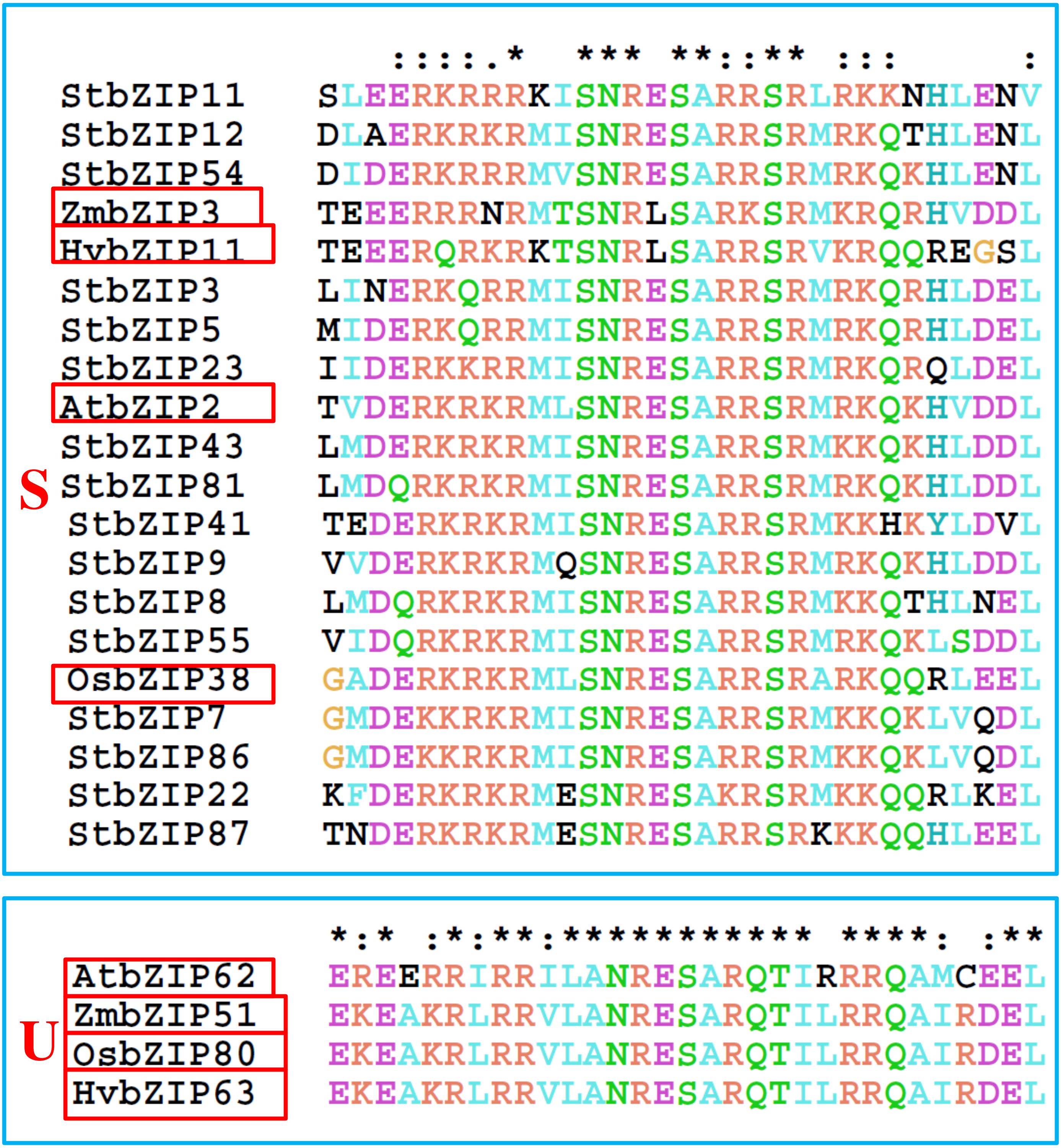

::::.*   *** **::** :::   :
StbZIP11 SLEERKRRRKISNRESARRSRLRKKNHLENV
StbZIP12 DLAERKRKRMISNRESARRSRMRKQTHLENL
StbZIP54 DIDERKRRRMVSNRESARRSRMRKQKHLENL
ZmbZIP3 TEEERRRNRMTSNRLSARKSRMKRQRHVDDL
HvbZIP11 TEEERQRKRKTSNRLSARRSRVKRQQREGSL
StbZIP3 LINERKQRRMISNRESARRSRMRKQRHLDEL
StbZIP5 MIDERKQRRMISNRESARRSRMRKQRHLDEL
StbZIP23 IIDERKKRRMISNRESARRSRMRKQRQLDEL
AtbZIP2 TVDERKRKRMLSNRESARRSRMRKQKHVDDL
StbZIP43 LMDERKRKRMISNRESARRSRMKKQKHLDDL
S
StbZIP81 LMDQRKRKRMISNRESARRSRMRKQKHLDDL
StbZIP41 TEDERKRKRMISNRESARRSRMKKHKYLDVL
StbZIP9 VVDERKRKRMQSNRESARRSRMRKQKHLDDL
StbZIP8 LMDQRKRKRMISNRESARRSRMKKQTHLNEL
StbZIP55 VIDQRKRKRMISNRESARRSRMRKQKLSDDL
OsbZIP38 GADERKRKRMLSNRESARRSRARKQQRLEEL
StbZIP7 GMDEKKRKRMISNRESARRSRMKKQKLVQDL
StbZIP86 GMDEKKRKRMISNRESARRSRMKKQKLVQDL
StbZIP22 KFDERKRKRMESNRESAKRSRMKKQQRLKEL
StbZIP87 TNDERKRKRMESNRESARRSRKKKQQHLEEL
: :*:**:*********** ****: :**
AtbZIP62 EREERRIRRILANRESARQTIRRRQAMCEEL
U
ZmbZIP51 EKEAKRLRRVLANRESARQTILRRQAIRDEL
OsbZIP80 EKEAKRLRRVLANRESARQTILRRQAIRDEL
HvbZIP63 EKEAKRLRRVLANRESARQTILRRQAIRDEL
