# Supplementary material for: Genome-wide identification and expression profiling of basic leucine zipper transcription factors following abiotic stresses in potato (Solanum tuberosum L.)
Source: PLoS One. 2021 Mar 12;16(3):e0247864. doi: 10.1371/journal.pone.0247864 (PMC7954325; doi:10.1371/journal.pone.0247864)
Supplement: S2 Table — The FPKM values were retrieved from the various transcriptomic datasets available at http://solanaceae.plantbiology.msu.edu/pgsc_download.shtml. (DOCX) [file pone.0247864.s003.docx]

**S2 Table. FPKM expression of *StbZIPs* in different tissues.** The FPKM values were retrieved from the various transcriptomic datasets available at http://solanaceae.plantbiology.msu.edu/pgsc_download.shtml.

| ***bZIP_ID*** | **Leaves** | **Roots** | **Tubers** | **Sepals** | **Petals** | **Fruit (Mesocarp & Endocarp)** |
| --- | --- | --- | --- | --- | --- | --- |
| *StbZIP1* | 5.32 | 41.17 | 49.00 | 14.03 | 18.33 | 19.96 |
| *StbZIP2* | 8.14 | 20.71 | 20.95 | 24.53 | 12.92 | 11.63 |
| *StbZIP3* | 0.00 | 9.55 | 1.24 | 1.11 | 0.00 | 7.86 |
| *StbZIP4* | 19.40 | 14.82 | 85.93 | 19.39 | 42.69 | 43.83 |
| *StbZIP5* | 0.00 | 0.59 | 0.15 | 0.00 | 0.00 | 0.00 |
| *StbZIP6* | 0.15 | 0.44 | 0.29 | 0.14 | 0.00 | 9.38 |
| *StbZIP7* | 0.14 | 5.70 | 0.00 | 1.46 | 5.36 | 3.30 |
| *StbZIP8* | 0.04 | 0.48 | 2.18 | 10.51 | 17.34 | 9.32 |
| *StbZIP9* | 5.86 | 14.86 | 6.46 | 8.92 | 11.30 | 32.74 |
| *StbZIP10* | 3.93 | 9.24 | 4.19 | 6.26 | 7.03 | 11.67 |
| *StbZIP11* | 0.20 | 1.30 | 0.43 | 2.34 | 0.33 | 1.24 |
| *StbZIP12* | 0.08 | 7.70 | 4.77 | 2.48 | 1.78 | 2.89 |
| *StbZIP13* | 4.27 | 12.77 | 10.76 | 13.15 | 7.73 | 8.59 |
| *StbZIP14* | 1.73 | 1.37 | 3.25 | 3.51 | 2.16 | 6.27 |
| *StbZIP15* | 9.30 | 24.88 | 4.44 | 14.37 | 5.16 | 2.53 |
| *StbZIP19* | 0.13 | 0.61 | 0.00 | 0.64 | 0.14 | 0.31 |
| *StbZIP20* | 20.27 | 40.30 | 49.39 | 52.23 | 44.41 | 66.96 |
| *StbZIP21* | 0.00 | 0.67 | 1.09 | 0.84 | 0.55 | 12.41 |
| *StbZIP22* | 15.01 | 44.97 | 67.21 | 28.80 | 33.43 | 14.90 |
| *StbZIP23* | 0.13 | 0.56 | 0.00 | 1.37 | 6.33 | 0.51 |
| *StbZIP24* | 0.00 | 0.59 | 0.00 | 0.45 | 7.64 | 0.10 |
| *StbZIP25* | 18.15 | 98.28 | 17.46 | 52.61 | 85.71 | 23.10 |
| *StbZIP29* | 0.00 | 1.78 | 0.00 | 0.67 | 2.07 | 0.03 |
| *StbZIP31* | 5.90 | 24.70 | 31.55 | 23.63 | 13.99 | 19.27 |
| *StbZIP32* | 0.00 | 0.68 | 0.17 | 0.00 | 0.00 | 9.15 |
| *StbZIP34* | 50.91 | 80.17 | 107.54 | 56.05 | 16.89 | 56.59 |
| *StbZIP37* | 0.07 | 8.28 | 0.00 | 1.00 | 26.05 | 1.31 |
| *StbZIP38* | 5.58 | 17.91 | 13.63 | 15.89 | 12.47 | 10.45 |
| *StbZIP40* | 3.17 | 4.13 | 2.06 | 19.12 | 5.71 | 3.29 |
| *StbZIP41* | 0.33 | 2.45 | 3.44 | 4.95 | 1.90 | 1.22 |
| *StbZIP42* | 4.54 | 45.86 | 58.38 | 26.68 | 13.28 | 98.79 |
| *StbZIP43* | 3.21 | 1.24 | 0.66 | 2.34 | 2.88 | 4.79 |
| *StbZIP45* | 1.79 | 3.66 | 2.32 | 5.69 | 4.32 | 1.52 |
| *StbZIP48* | 3.95 | 7.76 | 32.60 | 18.74 | 14.81 | 22.96 |
| *StbZIP49* | 27.01 | 14.85 | 19.18 | 35.18 | 29.43 | 18.32 |
| *StbZIP50* | 23.03 | 51.18 | 57.33 | 76.96 | 52.39 | 51.82 |
| *StbZIP53* | 10.28 | 8.50 | 12.37 | 20.72 | 17.83 | 12.28 |
| *StbZIP54* | 9.44 | 44.57 | 4.42 | 29.90 | 19.68 | 10.23 |
| *StbZIP55* | 15.21 | 1.77 | 11.75 | 1.66 | 0.70 | 2.59 |
| *StbZIP56* | 0.43 | 26.75 | 7.17 | 15.18 | 5.04 | 13.73 |
| *StbZIP59* | 0.00 | 0.13 | 0.46 | 0.00 | 0.00 | 0.28 |
| *StbZIP62* | 3.13 | 8.07 | 9.15 | 8.38 | 12.75 | 7.68 |
| *StbZIP63* | 18.54 | 29.98 | 61.20 | 73.48 | 48.44 | 23.48 |
| *StbZIP66* | 0.00 | 1.72 | 0.00 | 0.00 | 0.00 | 23.60 |
| *StbZIP68* | 13.59 | 35.29 | 58.95 | 28.88 | 22.29 | 30.03 |
| *StbZIP71* | 1.46 | 2.39 | 10.28 | 11.06 | 0.55 | 1.47 |
| *StbZIP74* | 19.65 | 37.55 | 42.77 | 34.08 | 27.31 | 23.44 |
| *StbZIP75* | 12.91 | 12.33 | 19.88 | 44.92 | 13.93 | 27.12 |
| *StbZIP76* | 4.73 | 19.80 | 19.83 | 13.88 | 8.73 | 15.27 |
| *StbZIP78* | 4.08 | 19.29 | 15.51 | 14.17 | 13.15 | 18.35 |
| *StbZIP80* | 7.12 | 12.22 | 7.63 | 10.98 | 6.19 | 6.96 |
| *StbZIP81* | 3.66 | 30.74 | 21.16 | 47.85 | 29.83 | 27.26 |
| *StbZIP87* | 13.65 | 29.00 | 44.59 | 28.86 | 30.59 | 252.62 |
| *StbZIP88* | 8.92 | 25.76 | 19.69 | 4.89 | 12.22 | 36.35 |
| *StbZIP89* | 74.49 | 151.85 | 421.66 | 281.51 | 247.33 | 201.72 |
|  |  |  |  |  |  |  |
