# Supplementary material for: Genome-wide identification and expression profiling of basic leucine zipper transcription factors following abiotic stresses in potato (Solanum tuberosum L.)
Source: PLoS One. 2021 Mar 12;16(3):e0247864. doi: 10.1371/journal.pone.0247864 (PMC7954325; doi:10.1371/journal.pone.0247864)
Supplement: S4 Table — (DOCX) [file pone.0247864.s005.docx]

S4 Table. Differential log 2 FC- FPKM, expression of *StbZIPs* in abiotic conditions (Salt-150 mM NaCl for 24h, Heat-35 °C for 24h, Mannitol-260 µM for 24h and water stress) with respect to control in potato plant at different time and concentrations.

| **bZIP_ID** | Salt – 150 mM NaCl, 24h | Heat – 24h, 35 °C | Mannitol – 260 µM, 24h | Water–Stressed Leaf |
| --- | --- | --- | --- | --- |
| *StbZIP1* | 0.28 | 2.12 | -0.36 | 2.59 |
| *StbZIP2* | -0.25 | -0.16 | 0.51 | 0.65 |
| *StbZIP3* | 0.64 | -2.48 | -0.13 | -3.42 |
| *StbZIP4* | -0.25 | -1.27 | 0.35 | -0.42 |
| *StbZIP5* | 0.86 | 6.99 | 1.04 | 0.00 |
| *StbZIP6* | -2.13 | 0.00 | 0.75 | 0.00 |
| *StbZIP7* | -0.02 | 3.45 | -1.02 | 1.92 |
| *StbZIP8* | 1.79 | 3.54 | -0.08 | -1.66 |
| *StbZIP9* | 0.45 | -0.12 | -1.08 | -0.62 |
| *StbZIP10* | 0.27 | 0.64 | 0.55 | -0.06 |
| *StbZIP11* | 1.74 | -1.04 | 1.45 | -0.66 |
| *StbZIP12* | 0.88 | 1.53 | 1.55 | 1.71 |
| *StbZIP13* | 1.17 | 1.87 | 1.46 | 1.56 |
| *StbZIP14* | 0.23 | -0.58 | 0.73 | 2.46 |
| *StbZIP15* | 0.62 | 2.60 | 0.48 | 0.69 |
| *StbZIP17* | 0.53 | 2.10 | 0.34 | 0.45 |
| *StbZIP19* | 1.41 | 5.52 | 1.15 | 0.00 |
| *StbZIP20* | 0.08 | 1.04 | 0.19 | 1.27 |
| *StbZIP21* | 1.79 | 0.96 | 3.05 | -0.08 |
| *StbZIP22* | 0.17 | -0.06 | -0.96 | 0.33 |
| *StbZIP23* | -0.60 | 5.83 | -1.66 | 0.75 |
| *StbZIP24* | 0.84 | -2.42 | 0.43 | 0.00 |
| *StbZIP25* | 0.57 | 1.80 | 1.00 | 2.73 |
| *StbZIP27* | 0.77 | -3.09 | -0.03 | 0.00 |
| *StbZIP29* | 0.14 | 0.99 | 0.66 | 0.74 |
| *StbZIP30* | 1.89 | 5.46 | 0.24 | 0.00 |
| *StbZIP31* | 0.65 | 0.06 | 0.17 | -0.77 |
| *StbZIP32* | -0.07 | -5.09 | -0.51 | 0.00 |
| *StbZIP34* | 0.54 | 0.48 | 1.22 | 1.39 |
| *StbZIP37* | 1.39 | 2.82 | 0.39 | 1.03 |
| *StbZIP38* | -0.60 | 0.71 | -0.46 | -4.05 |
| *StbZIP40* | 1.29 | 1.78 | 1.09 | 0.61 |
| *StbZIP41* | 1.90 | 3.11 | 1.66 | 0.17 |
| *StbZIP42* | 0.93 | 0.32 | 1.99 | 0.82 |
| *StbZIP43* | 1.30 | 0.33 | 1.90 | 0.26 |
| *StbZIP45* | 0.06 | 1.20 | 0.03 | -1.95 |
| *StbZIP48* | -0.09 | 0.34 | 0.60 | 0.17 |
| *StbZIP49* | -0.88 | -0.83 | -0.46 | 0.16 |
| *StbZIP50* | 0.11 | 0.47 | 0.84 | 0.98 |
| *StbZIP53* | 0.45 | -2.48 | 0.88 | 2.40 |
| *StbZIP54* | 1.36 | 0.63 | 2.28 | 3.91 |
| *StbZIP55* | 0.83 | 1.96 | 0.60 | -0.40 |
| *StbZIP56* | 0.55 | 1.29 | 0.79 | 1.67 |
| *StbZIP59* | 0.78 | 1.82 | 1.15 | 1.40 |
| *StbZIP62* | 2.13 | 6.01 | 1.64 | -2.47 |
| *StbZIP63* | 0.04 | 0.59 | 0.32 | 0.28 |
| *StbZIP66* | 2.35 | 3.37 | 3.71 | 0.07 |
| *StbZIP68* | 0.45 | 1.04 | 0.80 | 0.86 |
| *StbZIP71* | -0.43 | 0.57 | -0.67 | -2.22 |
| *StbZIP74* | 0.49 | -0.18 | 0.78 | -1.74 |
| *StbZIP75* | -0.27 | -0.23 | 0.24 | -1.54 |
| *StbZIP76* | 0.11 | -0.23 | 0.66 | 1.80 |
| *StbZIP78* | 0.77 | 1.09 | 1.27 | 1.82 |
| *StbZIP81* | 0.44 | -0.36 | 0.51 | 0.37 |
| *StbZIP82* | 0.51 | 2.51 | -0.27 | 3.75 |
| *StbZIP87* | 0.20 | -0.34 | -0.13 | 1.41 |
