# Supplementary material for: Genome-wide identification and expression profiling of basic leucine zipper transcription factors following abiotic stresses in potato (Solanum tuberosum L.)
Source: PLoS One. 2021 Mar 12;16(3):e0247864. doi: 10.1371/journal.pone.0247864 (PMC7954325; doi:10.1371/journal.pone.0247864)
Supplement: S5 Table — (DOCX) [file pone.0247864.s006.docx]

S5 Table. Differential log 2 FC-FPKM expression analysis after exogenous supply of phytohormones (BAP, ABA, IAA and GA) with respect to control in potato plant at different concentration and time course.

| **bZIP_ID** | BAP - 24h, 10 µM | ABA - 24h, 50 µM | IAA - 24h, 10 µM | GA3 - 24h, 50 µM |
| --- | --- | --- | --- | --- |
| *StbZIP1* | 0.20 | 2.27 | 0.07 | 0.85 |
| *StbZIP2* | 0.30 | 0.80 | 0.33 | 0.55 |
| *StbZIP3* | 0.28 | -0.80 | -0.29 | 0.00 |
| *StbZIP4* | 0.43 | -0.02 | 0.30 | 0.86 |
| *StbZIP5* | 0.48 | 0.00 | 1.07 | 0.91 |
| *StbZIP6* | 0.14 | 0.86 | -0.47 | 0.02 |
| *StbZIP7* | 0.41 | 2.25 | 0.93 | 0.51 |
| *StbZIP8* | 0.48 | -2.25 | 0.34 | 1.18 |
| *StbZIP9* | 0.26 | -1.16 | 0.31 | 0.34 |
| *StbZIP10* | 0.19 | 0.40 | 0.02 | 0.11 |
| *StbZIP11* | 0.65 | -0.17 | -0.25 | 0.25 |
| *StbZIP12* | 0.48 | 0.91 | -0.09 | 0.63 |
| *StbZIP13* | 0.34 | 0.00 | 0.09 | 0.14 |
| *StbZIP14* | 0.11 | 0.89 | 0.46 | 0.45 |
| *StbZIP15* | 0.30 | 0.24 | 0.09 | 0.28 |
| *StbZIP19* | 0.19 | -1.22 | -0.05 | 0.29 |
| *StbZIP20* | 0.23 | 0.27 | -0.15 | 0.09 |
| *StbZIP21* | 0.37 | -2.21 | -0.14 | -0.50 |
| *StbZIP22* | 0.40 | 0.04 | 0.30 | 0.24 |
| *StbZIP23* | 0.48 | 0.00 | -0.25 | 1.33 |
| *StbZIP24* | 0.11 | -2.56 | 0.01 | 0.22 |
| *StbZIP25* | 0.22 | 2.08 | -0.10 | 0.88 |
| *StbZIP29* | 0.13 | -2.52 | -0.09 | 0.12 |
| *StbZIP31* | 0.24 | 0.44 | 0.00 | 0.16 |
| *StbZIP32* | 1.90 | 1.33 | 1.49 | 1.16 |
| *StbZIP34* | 0.21 | 0.17 | 0.05 | -0.33 |
| *StbZIP37* | 0.08 | -3.14 | 0.32 | 0.47 |
| *StbZIP38* | 0.15 | 1.42 | 0.25 | 0.37 |
| *StbZIP40* | 0.26 | -0.34 | 0.03 | 0.60 |
| *StbZIP41* | 0.18 | -0.64 | -0.06 | -0.15 |
| *StbZIP42* | 0.33 | 0.73 | 0.63 | 0.86 |
| *StbZIP43* | 0.28 | 0.55 | -0.19 | 0.03 |
| *StbZIP45* | 0.27 | -0.52 | 0.09 | 0.15 |
| *StbZIP48* | 0.27 | -0.24 | -0.34 | -0.08 |
| *StbZIP49* | 0.21 | 0.84 | 0.01 | 0.01 |
| *StbZIP50* | 0.29 | 0.62 | 0.07 | 0.21 |
| *StbZIP53* | 0.12 | 0.33 | 0.28 | 0.11 |
| *StbZIP54* | 0.24 | -0.15 | -0.07 | 0.00 |
| *StbZIP55* | 0.15 | 0.69 | 0.13 | 0.72 |
| *StbZIP56* | 0.41 | 1.61 | 0.52 | 0.93 |
| *StbZIP59* | 0.32 | -0.67 | 1.07 | 1.41 |
| *StbZIP62* | 0.20 | 0.68 | -0.60 | -0.31 |
| *StbZIP63* | 0.26 | 1.53 | 0.14 | 0.20 |
| *StbZIP66* | 0.95 | 0.76 | 0.09 | 0.84 |
| *StbZIP68* | 0.25 | 0.96 | 0.08 | 0.04 |
| *StbZIP71* | 0.18 | 1.16 | 0.21 | -0.20 |
| *StbZIP74* | 0.22 | 0.55 | -0.07 | 0.17 |
| *StbZIP75* | 0.24 | 0.58 | 0.06 | 0.21 |
| *StbZIP76* | 0.29 | 0.15 | 0.38 | 0.23 |
| *StbZIP78* | 0.20 | -0.16 | 0.11 | 0.09 |
| *StbZIP80* | 0.27 | 0.61 | 0.22 | 0.30 |
| *StbZIP81* | 0.14 | 0.43 | -0.05 | -0.23 |
| *StbZIP87* | 0.50 | 0.85 | 0.42 | 0.13 |
| *StbZIP88* | 0.52 | 2.75 | 0.34 | 1.35 |
| *StbZIP89* | 0.40 | 0.30 | -0.08 | 0.06 |
