# Supplementary material for: Genome-wide identification and expression profiling of basic leucine zipper transcription factors following abiotic stresses in potato (Solanum tuberosum L.)
Source: PLoS One. 2021 Mar 12;16(3):e0247864. doi: 10.1371/journal.pone.0247864 (PMC7954325; doi:10.1371/journal.pone.0247864)
Supplement: S6 Table — (DOCX) [file pone.0247864.s007.docx]

S6 Table. List and primer sequence information of identified SSR of candidate StbZIPs

| Seq ID | Orientation | Start | Len | tm | GC% | Seq | Prod size | Seq len | Motif | Motif Len | SSR | SSR-Len |
| --- | --- | --- | --- | --- | --- | --- | --- | --- | --- | --- | --- | --- |
| StbZIP66 | FORWARD | 66 | 22 | 54.91 | 40.91 | CTTTCACTATGTGGTCATCAAG | 165 | 1069 | ATC | 3 | ATCATCATCATCATCATCATC | 21 |
| StbZIP66 | REVERSE | 230 | 20 | 54.54 | 40 | TCCATACTTCTTCCATGGTT |  |  |  |  |  |  |
| StbZIP7 | FORWARD | 378 | 20 | 54.28 | 40 | GAGAAATTTCAAGGTGGGTA | 136 | 2396 | TGATTT | 6 | TGATTTTGATTTTGATTTTGATTTTGATTT | 30 |
| StbZIP7 | REVERSE | 513 | 20 | 54.79 | 45 | GGAAATGGCAGAGATCATAG |  |  |  |  |  |  |
| StbZIP2 | FORWARD | 2 | 20 | 55.19 | 45 | GTGAGTTTGCACTTTTAGCC | 144 | 1410 | CCT | 3 | CCTCCTCCTCCT | 12 |
| StbZIP2 | REVERSE | 145 | 21 | 54.96 | 42.86 | AACTTCGGGTCCATAGTAAAC |  |  |  |  |  |  |
| StbZIP4 | FORWARD | 102 | 20 | 54.51 | 40 | CTTGTCATGAAATCCGTGTA | 147 | 2418 | AG | 2 | AGAGAGAGAGAG | 12 |
| StbZIP4 | REVERSE | 248 | 21 | 54.52 | 42.86 | TCCTCTATTCCTCTTTGTGTG |  |  |  |  |  |  |
| StbZIP4 | FORWARD | 1953 | 21 | 54.82 | 42.86 | TCAGCAGCTATCACTAAATCC | 152 | 2418 | CAA | 3 | CAACAACAACAA | 12 |
| StbZIP4 | REVERSE | 2104 | 21 | 55.38 | 42.86 | CATGCTTTGAAGTTGTAGAGC |  |  |  |  |  |  |
| StbZIP4 | FORWARD | 1617 | 21 | 55.79 | 42.86 | TGCAGAGATAGCTTTAGCAGA | 150 | 2418 | AAAG | 4 | AAAGAAAGAAAG | 12 |
| StbZIP4 | REVERSE | 1766 | 20 | 55.06 | 45 | GATAATGTGGTGGCTTCAGT |  |  |  |  |  |  |
| StbZIP4 | FORWARD | 2081 | 21 | 54.1 | 42.86 | ACAGCTCTACAACTTCAAAGC | 156 | 2418 | CATT | 4 | CATTCATTCATT | 12 |
| StbZIP4 | REVERSE | 2236 | 20 | 55.1 | 40 | GTGTTTTGTTGTGAATGCAG |  |  |  |  |  |  |
| StbZIP23 | FORWARD | 597 | 19 | 55.16 | 52.63 | CACCACTGCTCATCTCAAG | 152 | 771 | TGTT | 4 | TGTTTGTTTGTT | 12 |
| StbZIP23 | REVERSE | 748 | 20 | 55.93 | 40 | GACTCATTTGGCATTCATGT |  |  |  |  |  |  |
| StbZIP38 | FORWARD | 16 | 21 | 54.43 | 42.86 | GGTGTTTGAAGTTAGTGGAGA | 159 | 2220 | TAA | 3 | TAATAATAATAA | 12 |
| StbZIP38 | REVERSE | 174 | 19 | 54.95 | 57.89 | GGGCTGCAAGTCTACTACC |  |  |  |  |  |  |
| StbZIP38 | FORWARD | 356 | 21 | 54.9 | 42.86 | GGTGGAGACAGGAAAGTTTAT | 170 | 2220 | GGT | 3 | GGTGGTGGTGGTGGTGGT | 18 |
| StbZIP38 | REVERSE | 525 | 21 | 55.63 | 47.62 | CTGTTGAAGTGTCAGTCTGCT |  |  |  |  |  |  |
| StbZIP71 | FORWARD | 14 | 21 | 55.07 | 47.62 | GCTTCTCTGCCTTTATCCTAC | 152 | 1598 | CTTT | 4 | CTTTCTTTCTTT | 12 |
| StbZIP71 | REVERSE | 165 | 22 | 54.65 | 40.91 | AGCAATAACTGACCTTCTTAGG |  |  |  |  |  |  |
| StbZIP75 | FORWARD | 507 | 21 | 55.18 | 42.86 | GGTTGGGGTTAATAGTATTGG | 125 | 1991 | CTCAAG | 6 | CTCAAGCTCAAGCTCAAG | 18 |
| StbZIP75 | REVERSE | 631 | 21 | 55.62 | 47.62 | GAACCTCATCAAGAGTTAGGC |  |  |  |  |  |  |
| StbZIP25 | FORWARD | 179 | 21 | 54.46 | 47.62 | ATTAGGTGAGGACTGAGGAGT | 163 | 2135 | GTG | 3 | GTGGTGGTGGTGGTGGTGGTG | 21 |
| StbZIP25 | REVERSE | 341 | 22 | 54.28 | 40.91 | CAACGAATAGATAGATGACTGC |  |  |  |  |  |  |
| StbZIp | FORWARD | 260 | 21 | 54.47 | 47.62 | CTCTCTCTCCTCTCCTTGTTT | 150 | 1235 | ATACA | 5 | ATACAATACAATACA | 15 |
| StbZIp | REVERSE | 409 | 21 | 54.28 | 42.86 | CTTGCATTTTCCTCTACACTC |  |  |  |  |  |  |
| StbZIP54 | FORWARD | 149 | 21 | 55.58 | 42.86 | GTTCACTCCTTGGGAATGTAT | 139 | 1060 | GAA | 3 | GAAGAAGAAGAA | 12 |
| StbZIP54 | REVERSE | 287 | 21 | 56.15 | 47.62 | GGAGTCATCAGAACCTGAATC |  |  |  |  |  |  |
| StbZIP82 | FORWARD | 1302 | 21 | 55.3 | 42.86 | AGGACAAGCAAAAGAGGTAAC | 159 | 2148 | CAA | 3 | CAACAACAACAA | 12 |
| StbZIP82 | REVERSE | 1460 | 21 | 54.49 | 42.86 | AGCTTTGCTCCAGATGTACTA |  |  |  |  |  |  |
| StbZIP82 | FORWARD | 1723 | 21 | 55.05 | 42.86 | GCCACATTAGAAACTTGACTG | 145 | 2148 | TGTT | 4 | TGTTTGTTTGTT | 12 |
| StbZIP82 | REVERSE | 1867 | 21 | 55.86 | 42.86 | CCGAGACGAAGGATAATATGT |  |  |  |  |  |  |
| StbZIP5 | FORWARD | 30 | 21 | 55.66 | 42.86 | TGCAACAATCCACTACTTAGC | 149 | 738 | AAC | 3 | AACAACAACAAC | 12 |
| StbZIP5 | REVERSE | 178 | 22 | 54.98 | 40.91 | GAATAGGAAAAGAGAACGACTG |  |  |  |  |  |  |
| StbZIP41 | FORWARD | 845 | 21 | 54.78 | 42.86 | TCTGAGGAGAATGACGTGTAT | 162 | 1037 | ACCTT | 5 | ACCTTACCTTACCTT | 15 |
| StbZIP41 | REVERSE | 1006 | 20 | 54.57 | 40 | CTAATTTGTCAAAGGCCACT |  |  |  |  |  |  |
